# Body Perceptions and Psychological Well-Being: A Review of the Impact of Social Media and Physical Measurements on Self-Esteem and Mental Health with a Focus on Body Image Satisfaction and Its Relationship with Cultural and Gender Factors

**DOI:** 10.3390/healthcare12141396

**Published:** 2024-07-12

**Authors:** Mariana Merino, José Francisco Tornero-Aguilera, Alejandro Rubio-Zarapuz, Carlota Valeria Villanueva-Tobaldo, Alexandra Martín-Rodríguez, Vicente Javier Clemente-Suárez

**Affiliations:** 1 Faculty of Sports Sciences, Universidad Europea de Madrid, 28670 Madrid, Spain; nana_merino@hotmail.com (M.M.); josefrancisco.tornero@universidadeuropea.es (J.F.T.-A.); sandra.martin.rodriguez8@gmail.com (A.M.-R.);; 2 Faculty of Biomedical and Health Sciences, Universidad Europea de Madrid, 28670 Madrid, Spain; c.vtobaldo@gmail.com; 3 Grupo de Investigación en Cultura, Educación y Sociedad, Universidad de la Costa, Barranquilla 080002, Colombia

**Keywords:** body image satisfaction, social media influence, psychological well-being, cultural norms, gender differences, physical measurements

## Abstract

This narrative review examines the interplay among body image perceptions, social media influence, physical measurements, and their impact on psychological well-being, focusing on the roles of cultural and gender differences and the need to understand the research methodologies employed in this field. In the age of digital proliferation, platforms like Instagram and Facebook have reshaped body image concerns, often leading to increased dissatisfaction and psychological distress due to constant exposure to idealized images and a culture of social comparison. Physical attributes such as weight, height, and BMI are scrutinized under societal standards of health and attractiveness, contributing to a spectrum of mental health issues including low self-esteem, depression, and eating disorders. This examination reveals how cultural norms and gender expectations further complicate body image perceptions, affecting individuals differently based on societal and personal ideals. It synthesizes current research and types of methods to illuminate how these factors together influence mental health and self-esteem, advocating for comprehensive interventions and policy measures aimed at mitigating body dissatisfaction and promoting a healthier, more inclusive understanding of body image. By delving into the complexities of body image satisfaction and its psychological implications, this review highlights the necessity of addressing these concerns within public health and social policy frameworks, underscoring the importance of a multifaceted approach to enhance individual and societal well-being.

## 1. Introduction

In the expansive domain of contemporary psychological and sociocultural research, the scrutiny of body image and its profound impact on psychological well-being emerges as an increasingly critical area of scholarly interest [1]. As society grapples with the complexities of modern life, the interplay between individual self-perception and collective cultural narratives surrounding physical appearance assumes heightened significance [2]. This narrative review embarks on a meticulous exploration of the nuanced relationships among body image satisfaction, the pervasive influence of social media, and the significant psychological ramifications associated with adherence to, or deviation from, societal physical standards [3]. The analysis delves deeply into various factors including weight, height, and Body Mass Index (BMI), dissecting how these metrics collectively contribute to self-esteem and mental health, while also highlighting the distinct roles that cultural and gender differences play in shaping perceptions of body image across diverse populations [4]. In today’s digital era, marked by the dominance of platforms such as Instagram, Facebook, and TikTok, the landscape of body image concerns has undergone a significant transformation [5]. These platforms have evolved beyond mere venues for the display of idealized images; they now serve as active forums for social comparison, identity construction, and cultural dissemination. The continuous exposure to meticulously curated and often digitally enhanced portrayals of the human form fosters a culture of relentless comparison, leading to widespread body dissatisfaction and psychological distress among vast swaths of the global population [6].

This review aims to synthesize the current research, illuminating how these multifaceted factors collectively influence mental health and self-esteem and advocates for comprehensive interventions and policy measures aimed at mitigating body dissatisfaction and promoting a healthier, more inclusive understanding of body image. By examining the complexities of body image satisfaction and its psychological implications, this review underscores the necessity of addressing these concerns within the frameworks of public health and social policy, highlighting the imperative for a multifaceted approach to enhancing both individual and societal well-being. Additionally, it emphasizes the need to understand the research methodologies employed in the study of body image to inform effective intervention strategies.

### Methodology

To achieve our objective, we explored the primary and secondary literature, encompassing scientific journals, bibliographic repositories, and platforms such as PubMed, Scopus, Embase, Science Direct, Sports Discuss, ResearchGate, and the Web of Science. Our search leveraged terms compatible with body image and psychological well-being, including social media impact, body image satisfaction, self-esteem, mental health, cultural factors, gender differences, and physical measurements. Our narrative review spanned articles published from 1 September 2004 to 1 September 2024. 

We set specific exclusion parameters as follows: (i) works not aligned with the central theme of body perceptions and psychological well-being and (ii) doctoral theses, symposium summaries, and non-published materials. To ensure the thoroughness and relevance of our review, we initially identified a total of 450 articles through our extensive search across multiple databases. After applying our specific exclusion criteria and rigorous appraisal, we retained 280 articles that were directly pertinent to our research objectives. Then, after careful revision, 212 articles were included. This selection process underscores our commitment to providing a comprehensive and precise analysis of the impact of social media and physical measurements on self-esteem and mental health, with a focus on body image satisfaction and its relationship with cultural and gender factors. 

A panel of six reviewers diligently appraised the titles and summaries of all gathered documents to determine their relevance. Manuscripts utilizing outdated information, bearing topics not congruent with our research goals, or not written in English, were discarded. The same reviewers responsible for the paper selection independently extracted essential information from the chosen articles. The utilization of artificial intelligence tools was implemented during the figure creation process to enhance their visual appeal and increase their clarity for the reader. This included bing.com, a Microsoft-owned and operated search engine. Furthermore, AI-based translation tools were used as a supplementary measure to refine language. We upheld the highest standards of academic integrity and transparency, and the scientific content of our work (core research, analysis, interpretation, and writing) was conducted by the authors without the aid of AI and is entirely original and human-authored.

## 2. Theoretical Framework on Body Image

Understanding the implications of body perceptions on psychological well-being necessitates a foundational grasp of the theoretical frameworks that underpin body image research [7,8]. It is necessary to establish the definition of body image and body appreciation. Body image refers to an individual’s perception, feelings, and emotions around their own physical appearance. Positive body image literature identifies the latter as the primary factor, given its prevalence as a recurring theme in numerous qualitative investigations and its straightforward evaluative process [8]. Additionally, is useful to establish the concepts of body functionality [9]. According to Alleva et al., body functionality comprises everything the body can do or is capable of doing, including functions related to internal processes (e.g., catching a cold, digestion), physical abilities (e.g., walking, stretching), bodily senses and sensations (e.g., sight, experiencing pleasure), creative endeavors (e.g., drawing, singing), communication with others (e.g., through body language, eye contact), and self-care (e.g., sleeping, showering, etc.). On the other hand, body appreciation or body image is a complex and multidimensional construct that includes self-perceptions and attitudes (i.e., thoughts, feelings, and behaviors) regarding the body. It involves many individual yet related components, such as (but not limited to) appearance evaluation, appearance orientation, body esteem, and size perception accuracy [9]. Several key theories provide valuable insights into how body image relates to psychological health and the broader social and cultural contexts that shape these perceptions.

### 2.1. Objectification Theory

Objectification theory critically examines how societal practices contribute to the objectification of bodies, particularly those of women, and delineates the profound psychological consequences of such phenomena. This theory articulates that pervasive societal objectification leads individuals to self-objectify, adopting an observer’s perspective on their bodies [10]. This perspective not only alters how individuals perceive themselves but also manifests as a deep-seated societal issue where bodies are predominantly valued for their appearance or sexual appeal rather than their capabilities or individuality. The essence of this theory lies in its assertion that the internalization of an external viewpoint leads to habitual body monitoring [11]. This relentless self-surveillance consumes significant cognitive and emotional resources, detracting from one’s ability to engage fully in life’s broader aspects, such as personal achievements and relationships. The psychological toll is considerable, with individuals experiencing chronic shame and anxiety over their inability to meet culturally constructed beauty standards. These emotions can escalate to more severe mental health issues, including eating disorders and depression, and may also have somatic consequences, such as increased cardiovascular risk due to sustained stress and anxiety [12].

Critically, the theory highlights the central role of media and advertising in reinforcing body objectification. These platforms often promote narrow and unrealistic beauty standards, which are internalized by viewers, perpetuating cycles of objectification and self-surveillance [13]. While initially focused predominantly on the experiences of women, the principles of objectification theory are increasingly recognized as applicable across the gender spectrum. The rising incidence of objectified male images in media and advertising underscores that the psychological impacts of objectification are universally pertinent, necessitating a broadened scope of discourse and intervention [14]. Moreover, the impact of objectification varies significantly across different cultures and societies, shaped by local norms and values that influence beauty standards and the prevalence of objectification practices. Understanding these cultural nuances is crucial for crafting effective interventions and promoting a global movement toward body positivity [15]. In expanding the discussion around objectification theory, it is imperative not only to delve into its psychological impacts but also to challenge the societal norms and practices that sustain objectification. Efforts to promote diverse and realistic representations of bodies in the media, to foster environments where individuals are appreciated for their abilities and qualities beyond their physical appearance, and to encourage body positivity are vital steps toward mitigating the damaging effects of objectification [16]. These initiatives are essential for fostering a more inclusive understanding of body image issues and for promoting the well-being of individuals across diverse cultural and societal contexts.

### 2.2. Social Comparison Theory

Social comparison provides a comprehensive framework for understanding how individuals evaluate their self-worth and achievements relative to others. This evaluative process is critical across various dimensions of personal and social identity but holds particular significance for body image in the era of ubiquitous social media engagement [17]. The theory posits that individuals engage in both upward and downward comparisons, assessing themselves against others who they perceive as either superior or inferior in certain attributes, including physical appearance. In the context of body image, the dynamics of social comparison can be particularly impactful [18]. Upward comparisons judging oneself against those perceived as more attractive or fit can sometimes inspire motivation toward healthier behaviors or personal improvement. More commonly, however, these comparisons result in feelings of inadequacy and low self-esteem, compounded by the distorted perceptions of one’s physical self [19]. This is exacerbated by the unrealistic and often unattainable beauty standards portrayed in the media, which are magnified on social media platforms through a continuous stream of idealized and digitally altered images. Such relentless exposure can skew individuals’ perceptions of normalcy regarding body types and beauty standards, particularly affecting young and impressionable audiences. The result is an escalation in body dissatisfaction, closely linked to a range of mental health challenges including depression, anxiety, and eating disorders [20].

Moreover, social media introduces an additional layer of complexity to the social comparison dynamics through the quantification of social approval. Metrics like likes, shares, and comments provide tangible measures of social and personal worth, intensifying the focus on physical appearance in self-evaluation processes [21]. This scenario fosters a vicious cycle where individuals feel compelled to curate their online personas in ways that highlight physical attractiveness, perpetuating not only unrealistic standards but also emphasizing the importance of appearance in social valuation. Addressing the detrimental effects of social comparison theory on body image in the digital age necessitates multifaceted strategies [22]. Key among these is promoting media literacy, defined as the ability to access, analyze, evaluate, and create media in a variety of forms [23]. This includes educating users about the widespread use of photo editing and helping them recognize the selective nature of online sharing, which often excludes the realities of everyday life. Encouraging a culture of authenticity and diversity in media representations is equally crucial. It can help diminish the impact of harmful comparisons by broadening the spectrum of beauty that is celebrated in the media. Implementing educational programs and campaigns that emphasize the value of individual differences and advocate for a more inclusive definition of beauty could counter the dominance of narrow beauty ideals [24]. Furthermore, fostering resilience and self-esteem, especially among young people, is vital. These qualities can serve as buffers against the negative effects of unfavorable social comparisons, promoting healthier body image and enhancing overall mental well-being.

### 2.3. Self-Discrepancy Theory

Self-discrepancy theory offers a nuanced understanding of the psychological impacts resulting from the gaps among an individual’s perceived actual self, their ideal self (aspirations and hopes), and their ought self (duties and obligations). This theory is particularly salient in the study of body image, illustrating how significant discrepancies between an individual’s actual physical appearance and their idealized versions can lead to profound psychological discomfort [25]. At the core of this theory is the notion that these gaps are not just about aesthetic appearances or superficial standards; they represent deeper psychological processes that critically impact an individual’s self-esteem and mental health. The gaps often mirror societal and cultural beauty ideals, which are internalized by individuals and established as benchmarks. The pervasive influence of social media, advertising, and popular culture magnifies these ideals, often portraying them as attainable norms rather than exceptional outliers. As these images permeate individuals’ consciousness, the ideals become deeply entrenched, exacerbating the discrepancies between their actual and ideal selves, and thereby widening the chasm within their self-perception [25].

This internal conflict manifests as body image dissatisfaction, where feelings of shame emerge from harsh self-judgments based on these unmet internalized standards. Such emotional distress is persistent and can deteriorate into more severe conditions, affecting an individual’s sense of worth and competency. Additionally, the anxiety about failing to achieve or maintain these idealized body images can lead to social avoidance, disordered eating behaviors, or obsessive exercising, further impairing mental health and overall quality of life [26]. To address the psychological ramifications of self-discrepancy on body image, interventions often focus on promoting self-acceptance and challenging the unrealistic societal norms that fuel these discrepancies. Cognitive behavioral therapy (CBT), mindfulness practices, and positive psychology interventions are crucial techniques that help individuals reconcile their actual, ideal, and ought selves. These interventions foster a healthier body image and enhance general well-being by encouraging a more forgiving and accepting self-view. Moreover, broader societal changes are necessary to reduce the prevalence and impact of self-discrepancy related to body image. Promoting diversity in media representations of beauty, challenging stigmatizing attitudes toward body size and shape, and advocating for a more holistic view of health and well-being are vital. Educational programs and campaigns can be pivotal in changing societal norms that overemphasize physical appearance and undervalue other personal attributes [27].

By integrating objectification theory, self-discrepancy theory, and social comparison theory into both research and practical interventions, a comprehensive approach can be developed to tackle the multifaceted issue of body dissatisfaction and its mental health implications. This approach requires collaborative efforts across multiple disciplines, including psychology, sociology, media studies, and public health, to forge interventions that address the root causes of body image issues at societal, cultural, and individual levels.

## 3. Influence of Social Media on Body Image and Mental Health 

The rise of social media has profoundly transformed individual perceptions of body image, promoting a homogenized ideal of beauty and attractiveness that emphasizes narrow and often unattainable standards [3,4,6]. The widespread presence of social media (SM) has made it a critically important and relevant area of study, with significant consequences for both individuals and public health. Recently, experts and the general public have shown considerable interest in the difference between active and passive social media use. Engaging in active use, such as creating and sharing material and interacting with others, is often considered more beneficial. On the other hand, passive use, like simply surfing and scrolling, is often seen as either negative or having no particular impact. Recently, according to Valkenburg et al. (2021), the reason for increased passivity in social media was attributed to individuals experiencing mental health issues and depression [28].

Platforms such as Instagram, Facebook, and TikTok have become more than mere showcases for idealized images; they are arenas of intense social comparison where users continuously evaluate their worth against digitally enhanced portrayals of others [6]. This pervasive digital environment cultivates a culture of constant visibility and scrutiny, where body image is perpetually assessed against a backdrop of curated perfection. The consequences of this paradigm shift are significant, impacting body image satisfaction and, consequently, psychological well-being. Empirical studies reveal a complex relationship between social media engagement and body image concerns, demonstrating that higher levels of social media interaction are associated with increased body dissatisfaction, reduced self-esteem, and heightened anxiety about physical appearance. This trend is particularly pronounced among adolescents and young adults, who comprise a substantial segment of social media users and are at a pivotal stage in the development of their self-identity and body image [3,4,5,6].

The underlying mechanisms include exposure to unrealistic body standards and engagement in detrimental social comparisons. The internalization of appearance ideals, promoted through likes, comments, and shares, serves as quantifiable measures of social approval and personal value, reinforcing the significance of appearance in self-evaluation processes. The interactive nature of social media not only permits but also encourages users to seek validation for their physical appearance, fostering dopamine-driven feedback loops that underscore appearance as a critical source of self-esteem [29]. This dynamic can precipitate obsessive behaviors related to body monitoring, dieting, and exercising, which further entrench body dissatisfaction. Moreover, the anonymity and distance that digital platforms provide can sometimes lead to cyberbullying and body shaming, exacerbating the negative impact on individuals’ body images and mental health [30]. Despite these challenges, the relationship between social media and body image is not entirely detrimental. Some platforms and communities within the social media landscape have begun to challenge the prevailing tide of idealized images by promoting narratives of body positivity, diversity, and acceptance. These movements leverage digital platforms to advocate for a broader spectrum of beauty standards, celebrate diverse body types, encourage self-love, and confront the stigma associated with deviation from conventional beauty norms.

This emerging counternarrative highlights the potential for social media to serve as a catalyst for positive change, fostering healthier body image perceptions and enhancing overall psychological well-being. As such, understanding and navigating the influence of social media on body image requires a nuanced approach that recognizes both its detrimental and beneficial impacts.

### 3.1. Social Media as a Catalyst for Comparison

Social media platforms inherently cultivate an ecosystem ripe for social comparison, characterized by a relentless stream of polished, edited, and often unrealistic portrayals of life and physical appearance. This digital environment sets the stage for pervasive upward social comparisons, where users continuously measure their everyday realities against the idealized highlights of others. Such interactions intertwine self-evaluation with digital perceptions of beauty and success, profoundly impacting psychological well-being [31]. This phenomenon extends beyond mere dissatisfaction with one’s physical appearance—it affects how individuals perceive their overall worth, achievements, and social standing, amplifying traditional forms of social comparison. Thompson’s Tripartite Influence Model elucidates the mechanisms via which these relationships manifest. This concept posits that social actors, such as family, friends, and the media, develop the standards of physical attractiveness and slimness for women [32]. Additionally, the digital age has made these comparisons more immediate and accessible, simultaneously raising the standards against which individuals measure themselves to often unattainable heights. This incessant exposure does not merely alter self-perceptions but also skews the understanding of what is normal or achievable [5,6]. The blurring of lines between reality and digitally enhanced portrayals leads individuals to harbor unrealistic expectations for their bodies and lifestyles. The impact is especially pronounced among teenagers and young adults who are more susceptible to social media influences and are at a critical developmental stage concerning self-identity and body image [7,8].

Furthermore, the quantitative aspects of social media, such as likes, comments, and followers, add another layer of comparison. These metrics serve as tangible indicators of social approval and attractiveness, further entrenching the notion that self-worth is tied to physical appearance and online popularity. The pursuit of validation through these metrics can exacerbate feelings of inadequacy and propel individuals into a cycle of constant self-monitoring and modification of their online and physical selves to align with perceived standards of attractiveness [33]. The consequences of such dynamics extend beyond mere dissatisfaction, contributing to the increased prevalence of mental health issues such as depression and anxiety disorders. The link between social media-induced comparisons and mental health problems underscores the urgency of addressing this issue [34]. Strategies to mitigate these negative effects include promoting digital literacy to help individuals critically evaluate the content they consume, fostering online communities that celebrate diversity and authenticity, and encouraging a balanced approach to social media use that prioritizes real-world interactions and self-compassion over digital validation [35,36,37]. Understanding social media as a catalyst for comparison necessitates a critical examination of its structure and the content it promotes. It also calls for a concerted effort from individuals, communities, and policymakers to cultivate a digital environment that encourages positive social interactions and realistic portrayals of life and body image, thereby mitigating its potential harms and harnessing its capacity to connect and uplift [30].

### 3.2. The Role of Self-Presentation on Social Media

The role of self-presentation on social media significantly impacts how individuals perceive themselves and others, profoundly shaping the dynamics of body image and psychological well-being. As users navigate digital spaces, they frequently engage in selective self-presentation, a process where only the most flattering, joyful, or successful aspects of one’s life and appearance are shared. Driven by a desire to meet or exceed perceived societal standards of beauty, success, and happiness, this curated representation often starkly contrasts with individuals’ real-life experiences and self-perceptions. This phenomenon is not limited to the individuals curating their online presence; it also affects their peers who consume this content [38]. As users encounter these idealized portrayals, they may engage in upward social comparisons, measuring their own lives and bodies against an unrealistic standard that is nearly impossible to achieve. This cycle of comparison and dissatisfaction contributes to a broader cultural narrative around beauty and success that is increasingly disconnected from reality. Furthermore, the act of curating and maintaining an idealized online self demands constant vigilance and self-scrutiny, which can become a significant source of stress and anxiety. Users often find themselves preoccupied with capturing the perfect photo, achieving a particular look, or crafting an image that will garner approval and admiration from their social media peers [39]. This relentless pursuit of digital validation can detract from authentic self-expression and the enjoyment of real-life experiences, as moments are valued not for their intrinsic worth but for their potential as social media content.

**Figure 1:**
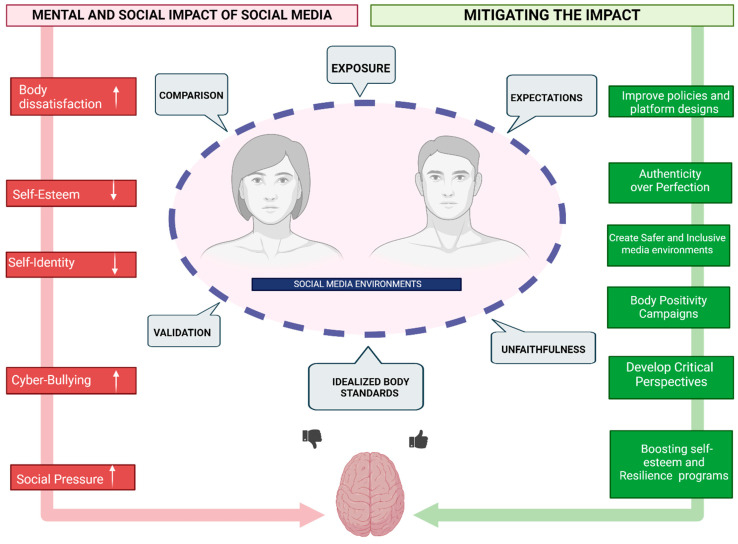
The influence of social media on mental health and potential strategies for intervention to address this issue; Up arrow: increase; Down arrow: Decrease.

The impact of this dynamic extends beyond the individual, influencing the broader social media culture and creating an environment where authenticity is obscured and appearances are paramount. This culture of comparison and performance can erode the sense of community and connection that social media platforms potentially foster, replacing it with competition and isolation. Addressing the challenges posed by self-presentation on social media requires a multifaceted approach. Educating users about the psychological impacts of selective self-presentation and encouraging more authentic and diverse representations of lives online can help mitigate these effects [40]. Platforms themselves can play a role by designing features that promote positive interactions and discourage harmful comparisons. Mental health interventions, such as cognitive behavioral strategies focused on building self-esteem and reducing reliance on external validation, can provide valuable support to those affected by these dynamics (Figure 1). Ultimately, fostering a digital environment that values authenticity over perfection, and well-being over appearances, is crucial for mitigating the negative impacts of self-presentation on social media. By promoting a culture of acceptance and diversity, individuals can navigate social media in ways that support their psychological health and foster genuine connections with others [41].

### 3.3. The Impact of Social Media on Adolescents and Young Adults

The influence of social media on adolescents and young adults is profound, significantly affecting their processes of self-identity and body image formation. This demographic, known for its high engagement with social media platforms, navigates a complex array of social expectations, idealized body standards, and the quest for identity in the digital age, highlighting the substantial impact of digital environments on young individuals’ self-perceptions and mental health [42]. Adolescents and young adults are particularly vulnerable to the effects of social media because of their developmental stage. As they strive to establish their self-identity, the inundation of idealized and often unattainable beauty standards on platforms such as Instagram, Snapchat, and TikTok can distort their perceptions of normalcy and attractiveness. This digital landscape becomes a battleground for self-esteem, where the pressure to conform to these standards can lead to profound dissatisfaction with one’s own body, potentially spiraling into disordered eating behaviors and distorted body image [43].

Moreover, the interactive nature of social media, with its system of likes, comments, and shares, introduces a quantifiable measure of social validation that is deeply intertwined with appearance. This dynamic can exacerbate the pressure to conform to idealized standards, as adolescents and young adults equate social approval and self-worth with how closely their bodies resemble those celebrated online [35]. The resulting decreased self-esteem and the insurmountable gap perceived between one’s real self and the polished images online foster feelings of inadequacy and worthlessness. The digital echo chambers created by algorithms can also intensify exposure to harmful content, including pro-anorexia and pro-bulimia messages, further endangering vulnerable individuals. The comparison trap is not limited to peer interactions but is amplified by celebrity and influencer culture, where the line between aspirational and normal becomes blurred, pushing young users toward unrealistic and harmful body goals [44].

Addressing these challenges requires a multifaceted approach that involves educators, parents, policymakers, and the platforms themselves. Educating adolescents and young adults about media literacy, encouraging critical engagement with social media content, and fostering open discussions about the pressures and unrealistic aspects of digital portrayals can empower them to navigate social media more healthily. The existing literature indicates that media literacy education helps young individuals develop critical thinking skills, enabling them to analyze and question the content they encounter on social media. This educational approach has been shown to reduce the negative impact of social media on body image and self-esteem [35,36,37,45]. Schools and communities can play a pivotal role in providing support systems and resources for young people struggling with body image issues [46].

Social media platforms also bear a responsibility to create safer, more inclusive environments that promote diverse body representations and provide tools to manage exposure to potentially harmful content (Figure 1). Legislative efforts to regulate the digital advertising of body standards and the promotion of extreme dieting or cosmetic procedures to minors can further safeguard their mental health. In essence, while social media has the potential to connect and inspire, its impact on adolescents and young adults concerning body image and self-esteem highlights a pressing need for a collective effort to mitigate its negative effects [47]. Through education, support, and regulation, it is possible to foster a digital landscape that supports the healthy development of self-identity and body image among the younger generation.

### 3.4. Cultural and Gender Differences in Social Media’s Impact

The impact of social media on body image and mental health varies significantly across cultural and gender lines, underscoring the complexity of these phenomena and the influence of societal norms and values in shaping individual responses to digital content. The interpretation and internalization of social media content are profoundly affected by cultural contexts, with some cultures emphasizing specific body ideals more strongly than others [48]. For instance, in societies where thinness is highly valorized, the widespread circulation of images showcasing slim figures on social media can exacerbate body dissatisfaction, leading to unhealthy eating behaviors and an increase in eating disorders [49]. Conversely, in cultures that celebrate fuller body types, social media narratives may pivot toward these ideals, yet still engender a sense of inadequacy among those who feel they do not meet these standards. Understanding the cultural lens through which social media impacts body image perceptions is critical for developing global interventions and support systems that are tailored to the specific needs of different cultural settings [50].

Gender differences also play a crucial role in how social media influences body image. Historically, the bulk of research has focused on the impact of social media on women, who are frequently targeted with images that promote and glorify thinness, leading to heightened body comparison and dissatisfaction [51]. This effect is compounded by the socialization of women in many societies to value appearance highly, rendering them particularly vulnerable to the influences of idealized images prevalent on social media platforms. Women’s experiences on social media are often characterized by intense scrutiny of physical appearance, which contributes to a higher incidence of body image concerns and related mental health issues [52]. However, the narrative that social media’s impact is confined to women is increasingly being challenged. Men, too, face their own pressures from idealized images on these platforms, with a growing body of research highlighting how social media shapes notions of idealized masculinity. Images that emphasize muscularity, leanness, and fitness can induce body dissatisfaction among men, pushing them toward extreme fitness routines and dietary behaviors in pursuit of an often unattainable physique. This recognition marks a critical expansion in the discourse around social media and body image, emphasizing the necessity for a gender-inclusive approach that acknowledges and addresses the pressures faced by all genders [53].

To address the diverse impacts of social media across different cultural and gender contexts, a profound understanding of the sociocultural dynamics at play is essential. Culturally sensitive research and interventions that account for the specific values, norms, and body ideals of different societies are required [54,55]. Moreover, promoting a broader representation of body types, gender expressions, and cultural backgrounds on social media can help mitigate the adverse effects associated with exposure to a narrow set of ideals. Creating a more inclusive and diverse digital environment not only reflects the reality of the global population but also empowers individuals to embrace their unique identities without conforming to a singular standard of beauty or attractiveness [56].

### 3.5. Mitigating the Negative Impact of Social Media

Mitigating the negative impact of social media on body image and mental health necessitates a comprehensive strategy that tackles the issue from various angles. Central to this approach is enhancing media literacy, which plays a critical role in empowering individuals to navigate the digital landscape more effectively. By educating users about the realities behind social media content, including the widespread use of filters, editing, and selective sharing, individuals can develop a more critical perspective on the images and narratives they encounter online. This increased awareness helps diminish the pressure to conform to unrealistic standards and reduces the intensity of social comparisons [57]. Promoting a balanced and healthy use of social media is another crucial component. Encouraging individuals to take regular breaks from social media, engage in digital detoxes, and maintain a robust offline life can help lessen the constant exposure to idealized images and the consequent feelings of inadequacy. This balance also involves curating one’s social media feeds to include a broader range of body types, lifestyles, and perspectives, thereby reducing the homogeneity in content that reinforces narrow standards of beauty and success [58]. Creating and fostering environments that celebrate body diversity and inclusivity is essential to counteracting the homogenized beauty ideals perpetuated by social media. This can be achieved by supporting platforms and creators that highlight diverse body types and stories, challenging the prevailing narratives around beauty and worth [36]. Campaigns and movements dedicated to body positivity and the normalization of all body types can significantly impact social media culture, making it a more welcoming space for all individuals, irrespective of their appearances. Interventions aimed at boosting self-esteem and resilience are also key to mitigating the adverse effects of social media (Figure 1) [59]. These interventions can include therapy, workshops, and programs designed to help individuals build a positive self-image and develop coping strategies for dealing with social media pressures. By strengthening self-esteem, individuals are better equipped to resist the detrimental impact of negative social comparisons and maintain a healthier relationship with their bodies [37].

Furthermore, the role of policymakers and social media platforms in this ecosystem is critical. There is a growing need for policies and platform designs that prioritize user well-being, including measures to combat cyberbullying, reduce the emphasis on physical appearance, and increase the visibility of diverse and realistic content [60]. Social media companies can leverage algorithms to promote content that fosters positive body image and mental health, creating a digital environment that supports rather than undermines well-being [61]. In conclusion, addressing the complex issue of social media’s impact on body image and mental health requires a concerted effort from individuals, communities, educators, mental health professionals, policymakers, and the platforms themselves. Through a multifaceted approach that includes promoting media literacy, encouraging balanced social media use, celebrating diversity, and fostering self-esteem and resilience, it is possible to cultivate a digital landscape that enhances rather than detracts from individual and collective well-being.

## 4. Impact of Physical Measurements on Psychological Well-Being

The correlation between physical measurements and psychological well-being is a nuanced and complex domain, highlighting the intricate interplay between individuals’ perceptions of their bodies and their mental health outcomes. These measurements are more than mere numbers; they represent a confluence of societal expectations, personal aspirations, and health indicators, playing a critical role in shaping an individual’s self-perception and emotional state [62]. In many societies, physical metrics are imbued with profound cultural and social significance, often construed as benchmarks for beauty, health, and even moral virtue. This cultural construction of body ideals exerts a significant influence on individuals, guiding their self-assessment and impacting their psychological health. The emphasis on specific body measurements can lead to critical self-evaluation against often unattainable societal standards. Such comparisons can engender feelings of inadequacy, lower self-esteem, and foster dissatisfaction with one’s body image, which, in turn, can adversely affect overall mental well-being [63].

Driven by the societal valorization of certain body types, individuals may enter a relentless cycle of dieting and excessive exercise, or even consider cosmetic procedures to align with these idealized metrics. Moreover, public discourse around weight, height, and BMI frequently intersects with health narratives, further complicating individuals’ relationships with these measurements [64]. While these metrics can provide valuable insights into general health status, their interpretation is often oversimplified, leading to stigma and discrimination against those who fall outside the perceived “normal” ranges. This stigma not only affects societal perceptions of individuals based on their physical measurements but also influences how individuals view themselves, potentially leading to a host of negative mental health outcomes, including anxiety, depression, and eating disorders [65]. The digital age, characterized by the omnipresence of social media, amplifies these issues, providing a platform for constant exposure to idealized body images and facilitating comparisons that exacerbate body dissatisfaction and psychological distress. Social media platforms often serve as echo chambers where societal beauty standards are reinforced, making it increasingly difficult for individuals to maintain a positive body image and psychological well-being in the face of pervasive messages valorizing certain body types over others [66].

Acknowledging the significant impact of physical measurements on psychological well-being needs a multidimensional approach that addresses the societal, cultural, and individual factors at play. This includes promoting body diversity and challenging the stigmatization associated with certain body types, encouraging a healthier, more inclusive understanding of beauty and health that recognizes the wide variability in human bodies [67]. Additionally, fostering critical media literacy to navigate the digital landscape with a more discerning eye can empower individuals to resist the pressures of conforming to narrow standards of beauty, thereby supporting healthier body perceptions and improved mental health outcomes [68].

### 4.1. Societal Standards and Their Psychological Impacts

Societal beauty standards, deeply embedded within cultural narratives and media representations, wield significant influence over individual self-perceptions and psychological well-being. These standards, which often prioritize thinness in women and muscularity in men, are propagated through various channels such as advertising, film, television, and, increasingly, social media platforms [69]. The pervasive reach of these ideals ensures that from a young age, individuals are exposed to and internalize specific criteria for beauty and attractiveness, criteria that are frequently unattainable for the majority of the population. The dissonance between societal ideals and the reality of natural body diversity creates fertile ground for body dissatisfaction and its psychological ramifications [70]. When individuals perceive a gap between their own physical measurements and the idealized forms celebrated by society, they may experience body dissatisfaction. This discontent does not exist in isolation; it is intricately linked to a host of psychological issues. Research consistently indicates that body dissatisfaction is a significant risk factor for low self-esteem, which can affect all aspects of an individual’s life, from personal relationships to professional achievements [71].

Moreover, the incessant pressure to conform to narrow beauty standards can lead to the development of serious mental health disorders. Conditions such as depression and anxiety can be exacerbated or triggered by persistent body dissatisfaction, creating a cycle of negative self-evaluation and emotional distress. The societal emphasis on physical appearance often leads individuals to base their worth on how closely they align with these external standards, neglecting other qualities and achievements [72]. Eating disorders represent one of the most severe consequences of societal beauty standards. The pursuit of thinness or muscularity can prompt destructive eating and exercise behaviors, as individuals strive to mold their bodies into the ideal shape. These disorders are complex mental health conditions with potentially devastating physical and psychological consequences, highlighting the dangerous extent to which societal standards can impact individuals [73]. Addressing the psychological impacts of societal standards requires a multifaceted approach. This includes challenging and diversifying the representation of beauty in media and culture to reflect the natural diversity of human bodies. Educational initiatives that promote body positivity and media literacy can empower individuals to critically engage with the images and messages they encounter, thereby reducing the impact of societal standards on self-esteem and body satisfaction [74]. Additionally, mental health support systems must be responsive to the challenges posed by societal beauty standards, offering support and treatment for those struggling with body dissatisfaction and its related disorders. By fostering a more inclusive and accepting societal view of beauty, it is possible to mitigate the psychological harm inflicted by narrow and unrealistic standards, promoting a healthier, more compassionate environment for all individuals [75].

### 4.2. Weight and Self-Esteem

**Figure 2:**
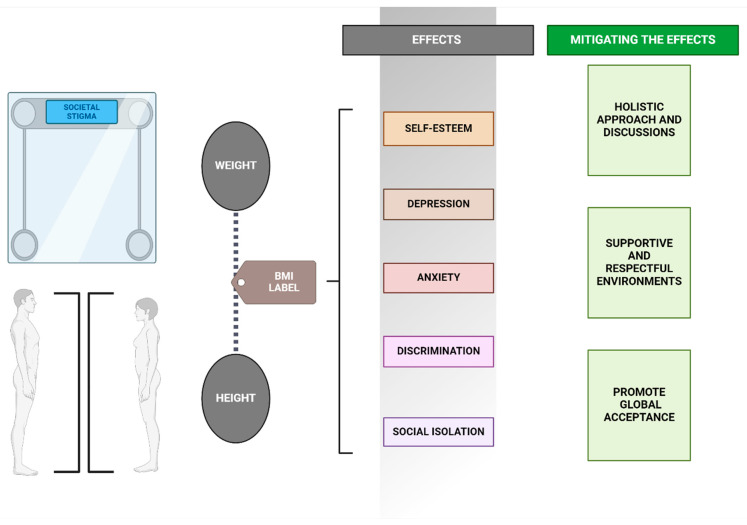
Effects that can be caused by the label marking weight and height measurements as well as BMI categorization.

Weight, as a highly visible and frequently scrutinized physical attribute, plays a central role in discussions about body image and self-esteem. Its impact on psychological well-being is profound, mediated by societal attitudes, stigmas, and the personal significance individuals attach to their body weight. The societal stigma associated with higher body weight is pervasive, manifesting in various forms of discrimination and prejudice that can infiltrate every aspect of life—from healthcare settings and employment opportunities to media representation and interpersonal relationships [76]. This stigma not only fuels body dissatisfaction but also catalyzes a spectrum of psychological issues, including lowered self-esteem, depression, anxiety, and social isolation (Figure 2). The relationship between weight and self-esteem is complex and bidirectional. On the one hand, societal pressures and internalized weight stigma can lead individuals to view their self-worth through the prism of their body weight, with deviations from the societal ideal negatively impacting their self-esteem [77]. On the other hand, low self-esteem can contribute to unhealthy behaviors related to weight management, such as disordered eating patterns, which may exacerbate weight concerns and further diminish psychological well-being. Conversely, being underweight is not free from societal judgment or psychological impact. The cultural valorization of thinness, particularly for women, complicates the experiences of individuals who are naturally or involuntarily underweight [78]. They may face assumptions about their health or eating habits, as well as pressure to conform to an idealized version of thinness that aligns with societal beauty standards. For men, being underweight can conflict with cultural ideals of masculinity that emphasize muscularity and physical strength, leading to feelings of inadequacy and diminished self-esteem [79].

Furthermore, the impact of weight on self-esteem is not limited to adult populations. Adolescents and young adults, who are navigating critical periods of identity formation and social comparison, are particularly vulnerable to the effects of weight stigma and body dissatisfaction [80]. The early internalization of negative attitudes toward weight can set the stage for lifelong struggles with self-esteem, body image, and mental health. Addressing the psychological impact of weight on self-esteem necessitates a multipronged approach that challenges societal weight stigma and promotes a more inclusive and compassionate understanding of body diversity [81]. Educational and awareness campaigns can play a crucial role in shifting societal attitudes, highlighting the harm caused by weight discrimination, and celebrating a range of body types. Healthcare professionals and mental health practitioners need to be cognizant of the role weight stigma plays in psychological distress, ensuring that their practices are informed by principles of body positivity and weight inclusivity [82]. Creating supportive environments, both online and offline, that affirm individuals’ worth irrespective of their weight is essential for fostering positive body image and self-esteem. This includes challenging and diversifying media portrayals of beauty, advocating for policies that protect against weight discrimination, and providing resources and support for individuals struggling with body dissatisfaction and its psychological repercussions [83]. Ultimately, decoupling self-esteem from body weight and challenging the societal norms that reinforce this link is crucial for promoting psychological well-being and a healthier relationship with body image. By embracing diversity and fostering a culture of acceptance and respect for all body types, society can mitigate the negative impact of weight on self-esteem and support individuals in achieving a positive and affirming sense of self [84].

### 4.3. Height, Body Image, and Mental Health

Height, much like weight, significantly influences individuals’ perceptions of their body image and, by extension, their psychological well-being. Societal valuation of height, particularly the preference for taller stature among men, is deeply embedded within cultural norms and stereotypes that associate height with attributes such as attractiveness, masculinity, and success [85]. This cultural construct creates a challenging dynamic for individuals, especially men, who do not meet these idealized standards, often leading to feelings of inadequacy, diminished self-esteem, and a negative self-perception. For men, the societal premium on taller stature extends beyond mere aesthetic preference; it intertwines with deeper societal perceptions of authority, strength, and desirability. The perceived shortfall in meeting these standards can result in significant psychological distress for shorter men, manifesting as lowered confidence, heightened self-consciousness, and, in some cases, social avoidance [86]. The impact of these societal biases can extend into various life domains, including romantic relationships, professional opportunities, and social interactions, where height is mistakenly equated with capability and charisma. Conversely, the discourse around height and its impact on women’s psychological well-being, while less pronounced, is nonetheless significant [87]. In certain contexts, taller women may experience feelings of self-consciousness or perceive themselves as less feminine, because of societal norms that idealize petite stature for women. Conversely, shorter women might feel overlooked or less imposing in professional and social settings, highlighting the complex ways in which height can influence self-esteem and body image across genders [88].

The impact of height on psychological well-being underscores the importance of context in shaping body image concerns. Cultural variations play a crucial role in determining the significance attached to height, with different societies and communities valuing different physical attributes. Regardless of these variations, the common thread is the link among height, societal expectations, and individual self-perception, a relationship that can significantly influence mental health outcomes [89]. Addressing the psychological impacts of height-related body image concerns requires a broader cultural shift toward embracing body diversity and challenging stereotypes associated with physical measurements. Promoting positive body image through education, media representation, and community support can help mitigate the negative effects of societal preferences for certain body types [90]. Furthermore, encouraging individuals to focus on their strengths and attributes beyond physical appearance can foster resilience and a more positive self-concept. Mental health interventions that specifically address body image concerns related to height can also be beneficial. Therapy and counseling can provide individuals with strategies to cope with societal pressures and build a healthier, more accepting relationship with their bodies. Support groups and online communities offer spaces for sharing experiences and strategies for overcoming height-related discrimination and bias [91]. Therefore, fostering a society that values individuals for their character, abilities, and contributions, rather than adherence to arbitrary physical standards, is crucial for improving body image perceptions and enhancing psychological well-being. By challenging the cultural narratives that link physical measurements like height to worth and success, it is possible to create a more inclusive and supportive environment for everyone, regardless of their stature [92].

### 4.4. The Complex Role of BMI

BMI is widely used to categorize individuals into weight-based classifications. Originally designed as a simple tool to assess potential health risks associated with body weight relative to height, BMI has evolved beyond its clinical origins to become a significant societal gauge influencing individuals’ body perceptions and their psychological well-being. By reducing complex physical attributes to simple numerical categories, BMI inadvertently fosters a culture of labeling and stigmatization, with profound implications for mental health (Figure 2) [93]. The categorization process, especially when individuals are labeled as overweight or obese, can precipitate a range of negative psychological outcomes. The stigma attached to these labels often leads to discrimination, shame, and social isolation, significantly impacting an individual’s self-esteem and body image. Such negative experiences can cultivate a hostile relationship with one’s body, affecting overall mental health [94]. Additionally, the anxiety associated with potential BMI classification can be particularly acute in settings where physical appearance is highly valued, such as during adolescence. Conversely, individuals categorized as underweight may face their own set of challenges, including societal pressure to conform to beauty standards that valorize thinness. This can validate harmful behaviors like disordered eating and discourage individuals from seeking necessary health interventions [95].

The reliance on BMI as a health assessment tool oversimplifies the diverse nature of health and body composition. Important factors such as muscle mass, bone density, and fat distribution are not accounted for in BMI calculations, potentially leading to misclassifications. Such inaccuracies can misrepresent an individual’s health status and lead to inappropriate health advice or interventions, undermining trust in health assessments and contributing to ongoing confusion and anxiety regarding personal health and body image [96]. Addressing the complex implications of BMI on psychological health calls for a more nuanced approach to how this metric is discussed and utilized. It is crucial for healthcare professionals and the broader society to acknowledge BMI’s limitations as a health assessment tool and to consider a more comprehensive view of an individual’s health, which includes mental well-being and lifestyle factors [97]. Promoting a holistic understanding of health that equally values mental and physical well-being is essential in mitigating the negative impacts of BMI categorization. Moreover, fostering a more inclusive and compassionate discourse around body image—one that challenges stigmatization and supports body positivity—can help counteract the detrimental effects of BMI-based judgments [98]. Educational efforts that emphasize the diversity of healthy body types and the limitations of using singular metrics like BMI as definitive health indicators can empower individuals to view their bodies through a lens of functionality and wellness rather than adherence to arbitrary standards [95].

Subsequently, re-evaluating the role of BMI in both healthcare and societal contexts is imperative for creating environments that encourage positive body image and enhance psychological health. By advancing toward a more individualized and comprehensive understanding of health, society can begin to dismantle the stigma associated with BMI categories and foster a healthier, more inclusive perspective on body diversity.

### 4.5. Addressing the Psychological Effects of Physical Measurements

Mitigating the negative psychological effects associated with physical measurements requires a multifaceted strategy that involves individual, societal, and systemic approaches. This comprehensive strategy not only aims to foster a healthier relationship between individuals and their bodies but also strives to challenge and reshape societal norms and beauty standards that amplify body dissatisfaction and related psychological issues [99]. At the heart of addressing these impacts is the promotion of body positivity. This movement advocates for acceptance of all body types, sizes, and shapes, challenging the narrow definitions of beauty that dominate societal discourse. By encouraging individuals to appreciate their bodies for functionality and uniqueness rather than adherence to arbitrary aesthetic standards, body positivity seeks to cultivate a more inclusive understanding of beauty. This involves enhancing diversity in media representations, fashion, and public discourse, thereby normalizing a broad range of body types and reducing the stigma associated with deviating from conventional beauty ideals [100].

Transforming societal beauty standards necessitates concerted efforts across various sectors, including media, education, and healthcare. Media outlets and content creators play a pivotal role by diversifying the body types and images presented to the public, moving away from homogeneous portrayals of beauty. Educational initiatives focused on media literacy can empower individuals to critically assess the images and messages they consume, building resilience against societal beauty pressures [101]. In healthcare, adopting a more holistic and compassionate approach to discussions about weight, BMI, and body image is crucial. Such an approach can help mitigate the stigmatization and anxiety often associated with physical measurements. By emphasizing comprehensive health over narrow metrics, healthcare providers can foster a supportive environment that respects body diversity [102]. Creating a culture that values individuals for their abilities, contributions, and character over physical appearance is essential for alleviating the psychological effects of physical measurements. This cultural shift involves challenging stereotypes and biases related to body size and shape, promoting respect and acceptance for all bodies, and emphasizing mental and emotional well-being as integral components of overall health [103]. Communities, workplaces, and educational settings can contribute to this cultural shift by implementing policies and practices that discourage discrimination and foster an inclusive environment. Psychological interventions, including therapy and counseling, are also vital. Approaches like cognitive behavioral therapy can offer effective strategies for improving body image and self-esteem [104]. These interventions help individuals challenge negative thought patterns related to their bodies, develop coping mechanisms for dealing with societal pressures, and cultivate a more positive and accepting self-perception. Support groups and online communities provide valuable spaces for sharing experiences and strategies for navigating body image concerns, offering social support, and reducing feelings of isolation. By collectively working toward a more accepting and diverse understanding of beauty, society can mitigate the negative impact of physical measurements on psychological well-being, paving the way for a healthier, more inclusive view of body image [105].

## 5. Body Image Satisfaction

Body image satisfaction is a crucial aspect of how individuals perceive and appreciate their body’s aesthetic and sexual attractiveness, serving as a fundamental component of self-esteem and psychological health. The perceptions individuals hold about their physical selves are deeply interconnected with their mental and emotional well-being, transcending superficial concerns to impact broader life domains [106]. This section explores the complex web of factors that influence body image satisfaction and its profound significance in individuals’ lives, along with its wide-ranging implications for mental health. The journey to achieving body image satisfaction is multifaceted, influenced by a confluence of personal, social, and cultural factors [107]. Media and popular culture propagate powerful images that shape societal expectations, while family and peer influences impart specific attitudes and values that further mold one’s body image. Internally, psychological factors such as self-esteem, personal experiences, and individual differences in temperament and resilience also play crucial roles in shaping how individuals perceive and feel about their bodies [108].

Societal norms and beauty standards set a backdrop against which individuals evaluate their physical appearance, often fostering a fraught relationship with body image. Recognizing the importance of body image satisfaction is essential, as it is intricately linked to overall psychological well-being [109]. Positive body image satisfaction can enhance self-esteem, bolster psychological resilience, and contribute to robust mental health. Conversely, dissatisfaction can lead to a spectrum of psychological issues, including depression, anxiety, and eating disorders. The implications of body image satisfaction extend into various facets of life, including social interactions, professional experiences, and personal relationships, influencing how individuals engage with the world [110]. Furthermore, body image satisfaction is not static but dynamic, susceptible to changes over time influenced by life experiences, aging, health changes, and shifts in societal attitudes toward beauty and health. This fluidity underscores the necessity of cultivating a positive and healthy relationship with one’s body, emphasizing acceptance, care, and respect over conformity to unattainable standards [111].

### 5.1. Factors Contributing to Body Image Satisfaction

Understanding the myriad factors contributing to body image satisfaction is crucial for navigating its complexities and formulating effective strategies to enhance it. This multifaceted phenomenon is influenced by a tapestry of social and cultural norms, personal experiences, psychological dispositions, and lifestyle choices, each playing a distinct role in shaping individuals’ perceptions and feelings about their bodies [112]. The powerful sway of media representations and cultural ideals that often propagate narrow definitions of beauty plays a significant role in shaping body image. These external influences include the images promoted by media and popular culture as well as attitudes and values imparted by family and peers. Additionally, internal psychological factors such as self-esteem, personal experiences, and individual differences in temperament and resilience significantly impact how individuals perceive and feel about their bodies [113].

Cultural norms and media representations act as powerful arbiters of beauty standards, often promoting specific body types, such as thinness in women and muscularity in men, as ideals. These widely propagated ideals are frequently unattainable for the average individual, leading to widespread dissatisfaction and feelings of inadequacy. The rise of social media has intensified exposure to idealized images, making these standards seem both ubiquitous and mandatory. Platforms like Instagram and Facebook not only feature curated content from celebrities and influencers but also encourage everyday users to present polished, edited versions of their lives and appearances [114]. Internally, an individual’s journey toward body image satisfaction is deeply influenced by personal and psychological elements. The feedback individuals receive from their immediate social environments—peers, family, and educators—can profoundly affect their body image. Positive reinforcement can enhance body satisfaction and self-esteem, while negative comments can lead to dissatisfaction and distorted self-perceptions [115]. Personal achievements that highlight the body’s functionality, such as sports or fitness milestones, can shift the focus from aesthetics to performance and utility, fostering a healthier body image. The role of physical activity and nutrition also highlights the dynamic interplay between physical well-being and psychological states. Engaging in regular physical activity and maintaining a balanced diet not only improves physical health but also enhances self-perception and overall psychological well-being [116]. These activities help individuals appreciate their bodies for their capabilities rather than just their appearance, potentially alleviating feelings of inadequacy brought on by unrealistic societal standards. To address body image satisfaction effectively, a comprehensive approach that includes promoting body diversity in media and society, encouraging positive interpersonal interactions, and supporting healthy lifestyle choices is essential [117]. Media literacy initiatives can empower individuals to critically engage with the content they consume, reducing the impact of harmful beauty standards. Additionally, fostering a supportive and inclusive community environment can help individuals feel valued and accepted regardless of their conformity to prevailing beauty norms [112].

### 5.2. Importance of Body Image Satisfaction

Body image satisfaction is a cornerstone of psychological well-being, serving as both a protective factor against mental health challenges and a promoter of a positive self-concept and life satisfaction [118]. The complex relationship between an individual’s perception of their body and their mental health is evident in the spectrum of psychological outcomes linked to either body satisfaction or dissatisfaction (Table 1).

Positive body image satisfaction is closely linked to a multitude of beneficial psychological outcomes that significantly enhance an individual’s quality of life. Individuals who are satisfied with their body image are less likely to experience pervasive feelings of sadness and anxiety, as a positive acceptance and appreciation of one’s body can buffer against stressors that exacerbate depressive symptoms and anxiety. Additionally, a healthy body image boosts self-worth and confidence, fostering a resilient and optimistic outlook on life that empowers individuals to pursue their goals and engage actively and positively in social interactions [119]. This satisfaction also extends to improving overall life quality, positively influencing physical health, social relationships, and personal fulfillment. Individuals content with their bodies are more inclined to adopt health-promoting behaviors, maintain fulfilling relationships, and pursue activities that bring joy and satisfaction. Moreover, a positive body image contributes to healthier and more fulfilling sexual relationships; comfort and confidence in one’s body can significantly enhance intimacy and sexual satisfaction, which are crucial for relationship contentment and personal well-being [120].

However, body dissatisfaction carries significant negative consequences that can severely impact an individual’s mental and physical health. It is a notable risk factor for developing eating disorders such as anorexia nervosa, bulimia nervosa, and binge eating disorder, where the pursuit of an idealized body image can drive individuals toward destructive eating behaviors, including excessive dietary restriction, bingeing, and purging. Additionally, chronic dissatisfaction with one’s body can lead to persistent sadness, low mood, and reduced self-worth, which may escalate into depression or chronic low self-esteem, severely affecting daily functioning and degrading quality of life [121]. Efforts to meet societal beauty standards or personal ideals often result in engaging in extreme dieting and excessive exercise, among other harmful practices, which not only endanger physical health but also perpetuate a cycle of dissatisfaction and negative self-perception. Furthermore, body dissatisfaction can cause individuals to avoid social interactions or situations where their bodies might be scrutinized, leading to social isolation, loneliness, and further deterioration in mental health. These diverse impacts highlight the profound effect that negative body image can have on overall well-being, necessitating interventions that address these issues comprehensively [122].

Addressing the intricate relationship between body image satisfaction and psychological well-being necessitates a holistic approach that includes promoting positive body image, supporting mental health, and challenging restrictive societal norms about beauty [123]. Creating environments that value diversity, encourage self-acceptance, and provide support for those grappling with body dissatisfaction can mitigate the adverse effects of negative body image and enhance individual well-being. By fostering communities that celebrate body diversity and offer robust support systems, society can significantly reduce the impacts of body dissatisfaction and cultivate a healthier, more inclusive perspective on beauty. This comprehensive approach not only improves individual outcomes but also enriches societal health and well-being [124].

### 5.3. Gender Differences in Body Image Satisfaction

**Figure 3:**
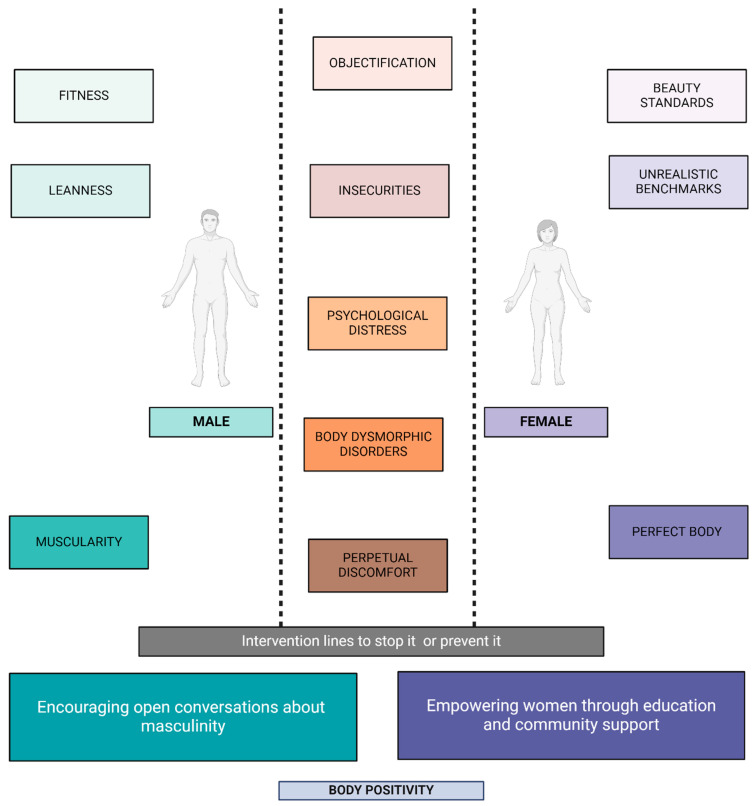
Differences between genders in body image perception solutions to mitigate the impact of satisfaction and strategies to address its influence.

Exploring gender differences in body image satisfaction reveals the intricate interplay among societal dictates, cultural imperatives, and individual psychological realms, offering insights into the distinct experiences that shape men’s and women’s perceptions of their bodies (Figure 3). This dichotomy is not just a reflection of individual struggles but also a manifestation of the broader societal and cultural landscapes that valorize specific body types, setting the stage for widespread body dissatisfaction [125]. For women, societal pressures to conform to an often unattainable standard of thinness are exacerbated by media representations that predominantly feature slender figures as the epitome of beauty and femininity. This constant exposure to a singular body ideal not only fosters an environment ripe for body dissatisfaction but also intertwines self-worth with physical appearance [126]. The psychological toll of navigating these expectations can lead to anxiety, depression, and unhealthy behaviors aimed at achieving the idealized body shape. Conversely, societal narratives for men typically emphasize muscularity, fitness, and ruggedness, portraying strength and physical prowess as hallmarks of masculinity. These ideals, perpetuated through media and cultural representations, establish challenging benchmarks that many men find difficult to meet [127]. Although the discourse on body dissatisfaction has historically centered on women, there is growing recognition of the pressures faced by men, leading to increased body dissatisfaction in this demographic as well. The pursuit of a muscular and lean physique can result in patterns of distress and disordered behaviors similar to those seen in women, underscoring the pervasive impact of societal standards on body image satisfaction across genders [128].

The psychological experiences underlying these gendered perceptions of body image are deeply personal yet universally influenced by societal and cultural expectations. The internalization of these ideals creates a complex psychological landscape where body dissatisfaction can diminish self-esteem, impact mental health, and alter interactions with the world [129]. Moreover, the stigma associated with failing to meet these standards can exacerbate feelings of inadequacy, leading to social withdrawal and a reduced quality of life. Navigating the terrain of body image satisfaction requires a nuanced understanding of the gender-specific pressures and challenges that individuals face [130]. It necessitates a critical examination of the societal and cultural constructs that define and uphold certain body ideals, alongside concerted efforts to challenge these norms and promote a more inclusive and diverse representation of bodies. Only by addressing these underlying factors can we hope to create an environment where body image satisfaction is accessible to all, unencumbered by gendered expectations and societal pressures [131].

#### 5.3.1. Women and Body Image Satisfaction

Societal pressures on women to conform to specific beauty standards are both pervasive and persistent, shaping their body image satisfaction through a complex matrix of cultural, media-driven, and interpersonal influences. The emphasis on thinness, youthfulness, and particular body proportions creates a narrowly defined ideal that is continuously amplified by media and advertising [132]. This omnipresent portrayal of idealized bodies sets an unrealistic benchmark for women, contributing to a societal milieu where deviation from these standards is often met with scrutiny and judgment. The role of media in shaping women’s body image is profound. From fashion magazines to social media platforms, women are bombarded with images that celebrate a specific body type, often digitally altered to achieve perfection [133]. This relentless exposure not only distorts perceptions of what is normal or attainable but also fuels a culture of comparison, where one’s self-worth becomes intricately tied to physical appearance. The impact of such comparisons can lead to widespread body dissatisfaction, prompting a cycle of negative self-evaluation and the pursuit of unhealthy behaviors aimed at achieving the elusive ideal [134]. The psychological toll of striving to meet these societal beauty standards is significant. Body dissatisfaction among women can lead to a host of negative emotional outcomes, including low self-esteem, depression, anxiety, and body dysmorphic disorders. These conditions profoundly affect women’s overall quality of life, influencing their social interactions, professional achievements, and personal relationships. The constant scrutiny and the pressure to conform can exacerbate feelings of inadequacy, leading to a state of perpetual discomfort with one’s physical self [135].

Adding to the challenge is the social scrutiny and commentary that women’s bodies often attract, both in public spaces and private conversations. This unsolicited feedback can range from direct criticisms to subtler, yet equally damaging, remarks and comparisons. Such commentary reinforces the notion that women’s bodies are open to evaluation and criticism, further entrenching body dissatisfaction and discomfort [136]. The experience of being observed and judged can also lead to social avoidance behaviors, as women may seek to escape the gaze and commentary that exacerbate their insecurities. Addressing the issue of body image satisfaction among women requires a multifaceted approach that challenges the societal norms and media practices contributing to the problem. Promoting diversity in media representation, advocating for body positivity, and challenging the cultural obsession with physical appearance are critical steps toward creating a more inclusive and supportive environment [137]. Empowering women through education and community support can foster resilience against the negative impacts of societal pressures. Psychological interventions, such as therapy and counseling, play a crucial role in helping women navigate body image dissatisfaction. By providing tools and strategies to combat negative self-talk, reshape perceptions of beauty, and build self-esteem, these interventions support women in developing a healthier relationship with their bodies. Ultimately, fostering body image satisfaction among women demands a collective effort to dismantle the deeply ingrained societal and cultural norms that dictate beauty standards [138]. By creating spaces that celebrate diversity and individuality, society can move toward a future where women feel valued and accepted, free from the constraints of unrealistic beauty ideals. Moreover, the phenomenon of objectification, where women’s bodies are viewed and valued primarily for their appearance or sexual appeal, adds another layer of complexity to female body image satisfaction. This objectification can lead to heightened self-surveillance, where women constantly monitor their bodies and engage in comparisons with others, reinforcing a cycle of dissatisfaction and self-criticism [139].

#### 5.3.2. Men and Body Image Satisfaction

The conversation around body image satisfaction is evolving, with an increasing recognition of the unique challenges men face. Societal expectations for men’s bodies, which emphasize muscularity, fitness, and leanness, have created a demanding cultural archetype. This “ideal” male physique, constantly reinforced by media and advertising, sets a standard that many find daunting and unattainable, leading to a growing prevalence of body dissatisfaction among men and challenging the stereotype that body image concerns are predominantly a female issue [140,141].

The portrayal of the ideal male body has undergone a significant transformation, with an increasing emphasis on muscularity and fitness. This shift reflects broader societal expectations and contributes to the internalization of these ideals among men. Constant exposure to images of sculpted, lean physiques across various media platforms sets unrealistic standards, causing many men to perceive their bodies as inadequate or inferior [141]. For some, achieving the idealized body becomes an all-consuming goal that dictates their lifestyle choices and behaviors. The pursuit of muscularity can drive men toward extreme and unhealthy behaviors, including excessive exercising that leads to overtraining, physical injury, fatigue, and neglect of other life responsibilities. The use of performance-enhancing drugs, such as steroids, presents serious health risks, including hormonal imbalances, liver damage, and increased risk of heart disease [142]. Disordered eating patterns, such as restrictive dieting, binge eating, or the use of laxatives and diuretics to control weight and body composition, jeopardize physical health and contribute to significant psychological distress. This obsession with achieving a certain body type can severely impact self-esteem, tying self-worth intricately to physical appearance and perceived bodily flaws. Conditions like body dysmorphic disorder, characterized by an obsessive focus on perceived physical defects, can severely impact mental health and daily functioning [143].

Addressing body image satisfaction among men requires a multifaceted approach that acknowledges the unique pressures they face. Promoting a broader, more inclusive representation of male bodies in media and advertising is crucial for challenging narrow definitions of male attractiveness. Education and awareness campaigns can help deconstruct societal expectations, encouraging men to appreciate their bodies for their functionality and strength rather than just their appearance [144]. Providing support for men struggling with body image concerns is also essential, including access to mental health services, support groups, and resources that address specific challenges in achieving body satisfaction. Encouraging open conversations about body image, masculinity, and vulnerability can help dismantle the stigma surrounding these issues, fostering a culture of acceptance and support. Ultimately, expanding the dialogue to include men’s experiences and challenges is vital for promoting healthier body image satisfaction for all genders [145]. By challenging societal expectations, promoting diversity, and providing support, it is possible to mitigate the negative impact of these pressures and empower individuals to develop a more positive and accepting relationship with their bodies. Understanding and addressing the gender differences in body image satisfaction requires a multifaceted approach that considers the unique pressures and challenges faced by both men and women [146]. For women, efforts to combat body dissatisfaction may involve challenging the societal valorization of thinness and promoting media literacy to critique and resist objectifying images. For men, destigmatizing discussions about body image and expanding the representation of male bodies in media to include a broader range of shapes and sizes can help mitigate the pressure to conform to unrealistic standards of muscularity and fitness [147]. Promoting a culture of body positivity, acceptance, and diversity is crucial for both genders, encouraging individuals to appreciate their bodies for their functionality and health, rather than solely for their adherence to societal beauty standards. This approach can foster greater body image satisfaction across genders, supporting individuals in embracing their bodies with confidence and self-compassion [148].

### 5.4. Cultural Influences on Body Image Satisfaction

Cultural influences on body image satisfaction weave a complex tapestry of beliefs, standards, and values that profoundly shape individuals’ perceptions and feelings about their bodies. Globally, diverse cultural contexts present a myriad of beauty ideals, each defined by unique preferences and expectations. These cultural standards dictate what is considered attractive within a community and significantly impact how individuals view their own bodies, influencing body image satisfaction positively or negatively [149]. The concept of beauty is deeply embedded in cultural narratives and histories and is far from universal. For example, many Western societies often valorize thinness, leading to widespread body dissatisfaction among those who do not meet this narrow criterion. Conversely, in some African, Caribbean, and Polynesian cultures, fuller body shapes are celebrated, with weight often associated with prosperity, health, and attractiveness [150]. In these environments, individuals conforming to the local standard of a fuller body shape may experience higher body image satisfaction, highlighting the significant influence of cultural norms. However, the impact of globalization and the spread of Western media has begun to shift traditional beauty ideals in many parts of the world. As Western standards of beauty become increasingly pervasive through media, social media, and advertising, they challenge and sometimes erode local cultural norms, leading to a homogenization of beauty standards. This shift often results in increased body dissatisfaction in cultures where Western ideals conflict with traditional standards of beauty [151].

In societies undergoing rapid modernization, the tension between traditional beauty standards and those introduced through globalized media creates a complex landscape for body image satisfaction. Individuals may find themselves caught between the expectations of their cultural heritage and the influential global norms, leading to confusion and conflict over body image. Specific cultural practices also significantly influence body image satisfaction. Rituals, ceremonies, and practices that celebrate the body in its natural form can foster a positive body image and an appreciation for physical diversity [152]. On the other hand, cultural practices that emphasize the modification of the body to meet certain standards can contribute to body dissatisfaction. Addressing the cultural influences on body image satisfaction requires promoting cultural sensitivity and awareness. Efforts to preserve and celebrate local beauty standards can help counteract the negative impact of globalized beauty ideals [153]. Educational and media literacy programs that encourage critical engagement with media representations of beauty can empower individuals to resist the pressure to conform to unrealistic standards. Additionally, fostering dialogues within communities about body image and cultural standards can contribute to a more inclusive understanding of beauty. By valuing diversity and challenging the notion of a singular beauty ideal, it is possible to create environments that support positive body image satisfaction across different cultural backgrounds. Recognizing and respecting the diversity of beauty standards across cultures is crucial in promoting a healthier and more inclusive view of body image satisfaction [154].

### 5.5. Enhancing Body Image Satisfaction

Enhancing body image satisfaction necessitates a holistic approach that integrates individual efforts with broader societal changes aimed at dismantling harmful beauty standards. The body positivity movement is pivotal, challenging narrow societal beauty ideals and promoting the acceptance of diverse body types through advocacy, inclusive media representations, and community support groups, thus fostering environments where all individuals feel valued [155]. Similarly, media literacy is crucial; educating individuals on the realities behind altered media images helps reduce their impact on body image satisfaction. Workshops and resources equip people to critically engage with media and appreciate natural body diversity. Mindfulness and self-compassion also serve as essential tools; practices like mindful eating and body gratitude exercises help develop a more accepting relationship with one’s body, promoting kindness toward oneself and counteracting negative self-comparisons [156]. For those experiencing significant distress, professional support such as cognitive behavioral therapy offers effective strategies for addressing deep-seated body image issues, helping to cultivate a healthier and more realistic body perception. On a societal level, advocating for diverse body representations in media, supporting antidiscrimination policies, and promoting a public discourse focused on health rather than appearance are vital [157]. These collective efforts encourage a societal shift toward body image satisfaction, highlighting the importance of inclusivity and support in overcoming the pervasive pressures of beauty standards. Ultimately, these strategies underscore the complex interplay between personal and societal factors in fostering a culture where diverse body types are accepted and celebrated, contributing to improved mental health and overall well-being [158].

## 6. Cultural Influences on Body Image

The intricate tapestry of body image perceptions is woven from a multitude of threads, each colored by the cultural contexts and gender norms prevalent within society. Far from being solely a personal matter, the way individuals perceive and feel about their bodies is significantly influenced by the collective beliefs, values, and expectations of the cultures they are part of. These cultural norms and gender expectations act as lenses through which body image is interpreted, often dictating the standards against which individuals measure their own bodies [159]. This exploration delves into the profound impact of these societal forces on body image perceptions, exploring the intricate ways in which cultural and gender-specific norms shape not only how individuals view themselves but also how these perceptions affect their overall psychological well-being. Cultural norms around beauty and body shape vary widely across different societies, with each culture espousing its own ideals of what is considered attractive [160].

These ideals are often perpetuated through various media, folklore, and social practices, embedding certain body image expectations deeply within the cultural psyche. Gender expectations also play a crucial role in molding body image perceptions. The dichotomy of societal roles and attributes traditionally associated with masculinity and femininity exerts a powerful influence, often leading to a gendered experience of body satisfaction or dissatisfaction. Moreover, the intersection of culture and gender norms presents a complex scenario where individuals navigate their way through overlapping expectations, striving to reconcile their personal body image with societal standards [161]. This navigation is fraught with challenges, as deviating from these prescribed norms can result in stigma, discrimination, or psychological distress. The implications of these cultural and gendered influences on body image are profound, affecting not only individuals’ self-esteem and body satisfaction but also their mental health and well-being. This exploration aims to shed light on the multifaceted relationship between cultural and gender norms and body image perceptions. By understanding the ways in which these societal forces influence body image, we can begin to unravel the complex dynamics at play, paving the way for more inclusive and compassionate approaches to fostering positive body image and psychological well-being across diverse cultural and gender landscapes [162].

### 6.1. Cultural Norms and Body Image

Cultural norms significantly shape standards of beauty and body ideals, deeply influencing individuals’ perceptions and satisfaction with their body image. These norms are dynamic and influenced by historical, social, and economic factors, which vary widely across different societies [163]. In Western cultures, the persistent valorization of thinness in women contributes directly to higher rates of body dissatisfaction and prevalent eating disorders. This emphasis on thinness is perpetuated through media, fashion, and entertainment, creating a pervasive culture where deviation from this ideal can result in stigma and a sense of inadequacy. Conversely, many non-Western cultures traditionally celebrate fuller body shapes as symbols of health, fertility, and prosperity, where a robust physique is associated with positive attributes, contributing to greater body image satisfaction among individuals [164].

However, globalization and the widespread dissemination of Western media have begun to challenge these traditional cultural norms. The global reach of Western beauty standards, facilitated by the internet, social media, and global advertising campaigns, has introduced a more homogenized view of beauty [165]. This globalization of beauty standards often represents a one-directional imposition of Western ideals, which can erode traditional cultural protections against body dissatisfaction. For instance, as Western media penetrate non-Western societies, the ideals of thinness and muscularity become global aspirations, impacting how individuals in these cultures perceive their bodies. This shift can lead to increased body dissatisfaction and related mental health issues in cultures where such problems were previously less prevalent [166].

Addressing the challenges posed by the globalization of body image standards while preserving cultural diversity and promoting body image satisfaction is crucial. Encouraging media literacy is a vital step, enabling individuals to critically assess the media they consume and recognize the diversity of beauty standards. There is also a growing need for media and advertising industries to adopt more inclusive and diverse representations that reflect global variance in body types and beauty ideals [167]. Furthermore, fostering cultural pride and appreciation for traditional beauty standards can serve as a bulwark against the uncritical adoption of homogenized, global beauty ideals. Community initiatives, educational programs, and the promotion of local art and literature can play vital roles in celebrating cultural diversity and reinforcing positive body image perceptions within these cultural contexts. By acknowledging and respecting the richness of cultural diversity in beauty standards, society can move toward fostering a more inclusive environment that supports positive body image satisfaction across the globe [168].

### 6.2. Intersectionality: Gender and Culture

The concept of intersectionality, which examines how various social categories such as gender, race, and culture interact on multiple levels to impact individual experiences, is crucial for understanding the nuanced dynamics of body image perceptions. By exploring the intersection of gender and culture, we uncover the complex ways in which these factors collectively influence body image satisfaction and dissatisfaction. This intersectionality reveals that the impact of gender on body dissatisfaction is not uniform across different cultural contexts but is instead shaped by the unique values and norms of each society [169]. For example, in cultures that prioritize communal values and collective well-being over an individual’s physical appearance, the gender gap in body dissatisfaction tends to be less pronounced. These societies often place less emphasis on personal aesthetics as a measure of worth, instead valuing individuals for their contributions to the community, their relationships, and their roles within the family. This cultural framework can provide a protective buffer against the negative impacts of body dissatisfaction, as the focus shifts away from individual appearance toward more holistic measures of value and identity [170].

Conversely, in societies characterized by individualism, where personal success, autonomy, and appearance are highly valued, the pressures to conform to idealized body standards are intensified. These individualistic values can exacerbate gender differences in body dissatisfaction, with women often facing more intense scrutiny regarding their appearance. The emphasis on personal achievement and the external validation of success can lead individuals, especially women, to internalize societal beauty standards more deeply, resulting in higher rates of body dissatisfaction and related mental health issues [171]. The role of media in shaping body image perceptions at the intersection of gender and culture is also significant. Media representations of beauty and body ideals are often filtered through the lens of cultural norms, further influencing how gender impacts body image dissatisfaction. In individualistic cultures, media portrayals that emphasize thinness for women and muscularity for men can reinforce the gender disparities in body dissatisfaction. However, in cultures with communal values, media that celebrate a broader range of body types and emphasize nonphysical attributes can help mitigate these disparities [172]. Understanding the intersectionality of gender and culture in body image dissatisfaction calls for tailored approaches that consider the specific cultural and gender-related factors at play. This includes developing culturally sensitive interventions that address the unique pressures and challenges faced by individuals at the intersection of these identities. It also involves advocating for more inclusive and diverse media representations that reflect the full spectrum of body types across different cultures and genders [173]. Furthermore, promoting education and dialogue around the importance of valuing individuals beyond their physical appearance, and recognizing the diverse ways in which beauty and worth are constructed across cultures, can contribute to reducing body dissatisfaction. By acknowledging and addressing the complex interplay between gender and culture in shaping body image, we can work toward a more inclusive and supportive society that fosters positive body image perceptions for all individuals [174].

### 6.3. The Impact on Psychological Well-Being

The intersection of cultural norms and gender expectations regarding body image casts a long shadow over individuals’ psychological well-being, with profound implications that ripple through various aspects of mental health. The pressures to align with idealized body images—a pursuit often fraught with unrealistic standards and unreachable goals—can significantly erode mental health, leading to a spectrum of psychological issues across diverse cultural backgrounds [175]. Body dissatisfaction, deeply rooted in the struggle to meet cultural and gender-specific ideals, emerges as a critical risk factor for various mental health challenges, including notably prevalent depression and anxiety. Individuals grappling with negative body perceptions may experience persistent sadness, hopelessness, or excessive worry about their appearance and how they are perceived by others. The constant internalization of idealized standards can lead to a pervasive sense of inadequacy, fueling depressive symptoms and anxiety disorders [176].

Disordered eating behaviors represent another significant impact of body dissatisfaction on psychological well-being. The drive to conform to specific body ideals can lead individuals down the path of restrictive dieting, binge eating, or other unhealthy eating patterns, which not only jeopardize physical health but also contribute to the development of eating disorders such as anorexia nervosa, bulimia nervosa, and binge eating disorder. These disorders entail severe psychological distress and can have long-term implications for mental and physical health [177]. The cultural context in which individuals reside plays a crucial role in shaping the standards of beauty and body image, thereby influencing the prevalence of body dissatisfaction and its psychological impacts. In societies where high value is placed on specific body types, individuals who perceive themselves as falling short of these ideals may experience heightened psychological distress. Gender expectations further compound these pressures, with women often facing intense scrutiny over their appearance and men dealing with expectations of muscularity and fitness [178].

Navigating these cultural and gender norms can be a source of significant stress, contributing to feelings of low self-esteem and inadequacy. The effort to align one’s appearance with societal expectations can lead to a constant state of dissatisfaction, where one’s self-worth becomes inextricably linked to physical appearance. This linkage fosters a vicious cycle of negative body image and psychological distress, where the failure to achieve idealized standards perpetuates feelings of inadequacy and further diminishes mental health [179]. Addressing the impact of cultural and gender-specific pressures on psychological well-being necessitates a comprehensive approach that includes promoting body positivity, fostering resilience, and providing support for those struggling with body dissatisfaction. Encouraging a more inclusive and diverse representation of bodies in media, challenging harmful beauty standards, and advocating for the decoupling of self-worth from physical appearance can help mitigate the psychological distress associated with body dissatisfaction [180]. Furthermore, providing accessible mental health resources, including counseling and support groups, can offer vital support to individuals navigating the challenges of body dissatisfaction. Educational initiatives aimed at building media literacy and promoting self-compassion can empower individuals to critically engage with cultural and media messages about body image, fostering a healthier relationship with their bodies and enhancing overall psychological well-being [181]. In summary, cultural and gender-specific pressures surrounding body image have significant implications for psychological well-being, necessitating a multifaceted response that addresses the root causes of body dissatisfaction and supports individuals in developing a positive and healthy body image.

### 6.4. Mitigating Cultural and Gender Influences

Mitigating the pervasive effects of cultural and gender influences on body image satisfaction requires a comprehensive and nuanced strategy that recognizes the complexity of these issues. A multifaceted approach that includes promoting cultural diversity, implementing gender-sensitive interventions, advancing education and advocacy, and fostering supportive communities is essential for addressing the root causes of body dissatisfaction and fostering a more positive body image landscape [182].

Embracing and celebrating cultural diversity in body types and beauty standards is crucial in dismantling the monolithic ideals that dominate current societal narratives. This involves showcasing a wide range of body shapes, sizes, and appearances in media and advertising, as well as elevating voices and stories from diverse cultural backgrounds to challenge the hegemony of Western beauty standards [183]. By valuing and highlighting the richness of body diversity, individuals can find affirmation and representation, reducing the pressure to conform to a narrow set of ideals. Tailored interventions must address the specific challenges and concerns faced by each gender. For women, combating the societal obsession with thinness and youth is critical, while for men, the focus might be on challenging norms around muscularity and physical strength. Gender-sensitive programs that address these distinct needs can help individuals navigate body image issues more effectively, promoting resilience and a healthier relationship with their bodies [184].

Educational initiatives that foster critical thinking about the cultural and social construction of beauty standards are vital. Advocacy efforts aimed at challenging and changing these standards can empower individuals to resist the pressure to conform to unrealistic ideals. Educational programs, both within schools and community settings, can provide tools for understanding and critiquing how beauty standards are propagated and internalized. Creating supportive communities that affirm diversity in body types and provide safe spaces for individuals to share their concerns and experiences with body image is crucial for promoting well-being [152]. These communities can be online forums, social media groups, or in-person support groups and counseling services, offering solidarity, understanding, and practical strategies for navigating body image concerns. This contributes to more positive and affirming environments for all. In conclusion, effectively mitigating the impact of cultural and gender influences on body image requires a holistic approach that combines the celebration of diversity, tailored interventions, education, and robust support systems. By acknowledging the complexity of these influences and actively working to challenge and change harmful norms, society can foster a more inclusive and positive environment that supports the psychological well-being of all individuals, regardless of their cultural background or gender identity [185].

## 7. Impact of Body Image on Psychological Well-Being

The intricate relationship between body image and psychological well-being is a critical concern within mental health discourse. As individuals navigate the complexities of their daily lives, their perceptions of their bodies significantly influence a wide range of psychological outcomes. Body image satisfaction—or the lack thereof—profoundly impacts one’s emotional state, behavioral patterns, and overall quality of life, setting a foundation for either psychological resilience or vulnerability [166]. This connection underscores the importance of addressing body image concerns not merely as issues of vanity but as substantive contributors to mental health. Body image satisfaction can serve as a protective factor, bolstering self-esteem, fostering positive social interactions, and promoting healthy lifestyle choices. Conversely, body image dissatisfaction can trigger a cascade of negative psychological effects, including heightened stress, withdrawal from social activities, and risky behaviors aimed at altering one’s physical appearance. These psychological states can extend far beyond transient moods, potentially influencing long-term mental health and well-being [48].

### 7.1. Relationship between Body Image Satisfaction/Dissatisfaction and Mental Health Outcomes

The relationship between body image satisfaction or dissatisfaction and mental health outcomes is pivotal for psychological well-being, affecting individuals’ emotional, cognitive, and social functioning [186]. This complex, bidirectional relationship underscores the importance of nurturing a positive body image as a fundamental aspect of mental health care.

Body image satisfaction enhances psychological state, boosts self-esteem, and fosters resilience, contributing to effective stress management and a positive life outlook. This satisfaction correlates with reduced symptoms of depression and anxiety, serving as a protective barrier against these common mental health issues. Furthermore, individuals content with their body image tend to have better social relationships, engage more in health-promoting behaviors, and experience greater personal fulfillment [187]. Conversely, body image dissatisfaction poses significant risks, often leading to negative psychological outcomes. The distress from not matching culturally endorsed body ideals can result in persistent self-criticism and emotional turmoil, undermining self-esteem and increasing depressive symptoms and anxiety. Such dissatisfaction may limit social participation, reduce relationship satisfaction, and lead to avoidance of body-centric activities [188]. Additionally, severe mental health conditions like eating disorders and body dysmorphic disorder are closely linked to body dissatisfaction. Eating disorders such as anorexia nervosa, bulimia nervosa, and binge eating disorders arise from extreme body dissatisfaction and the compulsion to achieve a particular body type, necessitating comprehensive treatment. BDD involves an obsessive focus on perceived appearance flaws, causing significant distress and functional impairment [189]. Understanding this relationship highlights the need for body image interventions as part of mental health care. Promoting body positivity, challenging societal beauty standards, and providing support for those struggling with body image issues are crucial. Such initiatives can greatly mitigate the negative impacts of body image dissatisfaction and support the development of a societal environment that encourages mental and emotional well-being, free from the damaging effects of negative body perceptions [190].

### 7.2. Psychological Disorders Associated with Poor Body Image

The intricate connection between poor body image and various psychological disorders highlights the critical need for comprehensive mental health care that addresses body image concerns. Disorders commonly linked with poor body image include eating disorders, mood disorders, and body dysmorphic disorder, each presenting unique challenges and necessitating tailored interventions [191].

Eating disorders such as anorexia nervosa, bulimia nervosa, and binge eating disorder are directly tied to body image dissatisfaction. These conditions stem from a distorted perception of body weight and shape, where individuals engage in harmful behaviors like extreme dieting, purging, and binge eating. Driven by profound fears of weight gain or a desperate need to lose weight, these disorders are severe manifestations of the internalization of unrealistic body standards. Effective treatment requires addressing both the behavioral symptoms and the underlying body image issues to ensure sustainable recovery [192].

Mood disorders, particularly depression and anxiety, are closely associated with poor body image. Individuals suffering from negative body perceptions may experience ongoing sadness, loss of interest in previously enjoyed activities, and a pervasive sense of worthlessness—classic symptoms of depression. Anxiety, especially social anxiety, can intensify because of fears of appearance-based judgment or rejection, leading to social withdrawal and exacerbating depressive and anxious symptoms. Furthermore, body dysmorphic disorder represents an extreme concern for body image, where individuals obsess over perceived or minor flaws in their appearance, which are often imperceptible to others. BDD can cause significant distress and impairment in daily functioning, avoidance of social interactions, and in severe cases, suicidal ideation [193]. Effective management of BDD involves recognizing its roots in body image issues and applying targeted psychological interventions. To address the psychological disorders associated with poor body image comprehensively, mental health practitioners must employ a multifaceted approach. This includes cognitive behavioral therapy to correct distorted perceptions of the body, psychoeducation to counteract harmful societal beauty norms, and support groups that offer a supportive community environment. Promoting body positivity and resilience against negative cultural and media influences is also essential in preventing the development of related psychological disorders. By integrating body image interventions into broader mental health care, clinicians can offer more holistic support, aiding individuals in not only overcoming psychological disorders but also cultivating a healthier, more positive relationship with their bodies [194].

### 7.3. Addressing the Impact of Body Image on Psychological Well-Being

Addressing the profound influence of body image on psychological well-being demands a comprehensive, multifaceted approach that targets both individual perceptions and broader societal factors. This strategy should integrate educational initiatives, media literacy, targeted mental health interventions, and community support to create a more inclusive society that fosters positive body image perceptions. Education is fundamental in shaping healthier body image perceptions. Initiatives targeting all age groups, particularly from young ages, are vital [195]. Such programs should educate about body diversity, physiological changes during puberty, and the manipulation behind many media portrayals of beauty. Equipping individuals with the skills to critically evaluate and challenge societal beauty standards empowers them to develop a more accepting view of their bodies. Media literacy is equally critical, helping individuals discern the realities behind heavily edited media images, thereby mitigating the impact of negative media influences on body image [195]. For those battling significant body image dissatisfaction, mental health interventions like cognitive behavioral therapy, acceptance and commitment therapy, and dialectical behavior therapy can be effective. These therapies help individuals manage distressing thoughts and emotions related to body image, enhancing psychological resilience [45].

Furthermore, advocating for more diverse representations in media and fashion, supporting anti-weight discrimination policies, and establishing community spaces for shared experiences are crucial. These actions not only promote diversity but also contribute to a societal shift toward more realistic beauty standards [196]. Community support systems provide essential platforms for reassurance and mutual support, helping individuals navigate body image challenges and enhancing overall well-being. In summary, a holistic approach to addressing body image issues involves concerted efforts across educational, clinical, societal, and community levels. By implementing strategies that encourage body positivity, challenge outdated norms, and provide robust support, society can better support individuals in their journey toward acceptance and foster an environment conducive to psychological well-being [197].

### 7.4. The Importance of Body Image in Self-Evaluation and Its Psychological Impact

Understanding the role of body image in an individual’s self-evaluation is crucial for comprehensively addressing its impact on psychological well-being. People vary widely in the extent to which they consider their body image as a significant component of their overall self-evaluation. This variation can significantly influence their mental health outcomes.

Individuals who place high importance on body image as a core aspect of their self-evaluation often experience more pronounced psychological effects related to body image satisfaction or dissatisfaction. For these individuals, their sense of self-worth and confidence is closely tied to their physical appearance. As a result, body image dissatisfaction can lead to heightened emotional distress, lower self-esteem, and increased vulnerability to mental health disorders such as depression, anxiety, and eating disorders. The pressure to meet societal beauty standards can exacerbate these effects, leading to a persistent cycle of self-criticism and negative emotional states [189,193]. Conversely, individuals who view body image as only one small piece of their overall self-evaluation tend to exhibit greater psychological resilience. For these individuals, self-worth is more likely derived from a broader array of attributes, including personal achievements, relationships, and intrinsic qualities such as intelligence, kindness, and creativity. This diversified self-evaluation framework provides a buffer against the negative impacts of body image dissatisfaction. While they may still experience some level of concern about their appearance, it does not dominate their overall self-perception, allowing them to maintain better psychological health and emotional stability [187].

Promoting body neutrality, as discussed previously, aligns well with this approach. By shifting the focus from appearance to functionality and capability, individuals can develop a healthier relationship with their bodies, grounded in an appreciation for what their bodies can do rather than how they look. This perspective not only supports better mental health outcomes but also fosters a more inclusive and accepting societal attitude toward body diversity.

## 8. Methodological Approaches in Body Image Research

The study of body image, encompassing individual perceptions, emotions, and attitudes toward one’s physical appearance, necessitates a multifaceted methodological approach because of its complex nature. This complexity arises from the diverse factors influencing body image, including cultural and gender norms, the pervasive impact of social media, and individual physical characteristics. To dissect the extensive influences on body image and its consequential effects on psychological well-being effectively, researchers employ a variety of methodological strategies, reflecting the necessity for academic rigor and adaptability in their investigative methods [4].

These methodological approaches facilitate a comprehensive exploration of how body image perceptions are formed, sustained, and altered across different cultural contexts and among diverse demographic groups. They also enable researchers to delve into the psychological effects of body image dissatisfaction, such as increased susceptibility to mental health disorders, erosion of self-esteem, and diminished overall quality of life [104]. Highlighting the primary research methods used in this field, including quantitative surveys, experimental designs, qualitative interviews, and focus groups, this discourse not only points out the strengths and potential biases of these approaches but also stresses the need for nuanced, multidimensional analysis. Such an approach is crucial for advancing our understanding of the intricate relationship among individual body image perceptions and the broader psychological, cultural, and social frameworks that shape these perceptions, thereby enhancing our ability to address and mitigate the negative impacts of body dissatisfaction [198].

### 8.1. Quantitative Research Methods

In the field of body image research, quantitative methods such as surveys, questionnaires, and experimental studies are instrumental in elucidating the complexities of how individuals perceive their bodies and the subsequent psychological impacts. Surveys and questionnaires are essential for capturing a wide array of data on body image satisfaction or dissatisfaction, concerns about specific body parts, and the psychological effects linked to these perceptions, including impacts on self-esteem and mental health issues like depression and anxiety [199]. These tools enable researchers to gather large-scale data from diverse populations, offering a macroscopic view of body image trends and patterns. However, the reliability of these methods can be compromised by factors such as social desirability bias, where participants may modify their responses to align with perceived social norms, and inaccuracies in self-perception that can skew the results. Despite these challenges, surveys and questionnaires remain invaluable for identifying broad trends and informing further qualitative research and intervention strategies [200]. Experimental studies, on the other hand, provide insights into the causal relationships between specific variables and body image perceptions. By manipulating variables within a controlled setting, researchers can isolate the effects of certain stimuli—such as exposure to idealized media images—on body image satisfaction [201]. These studies are critical for confirming causality and are fundamental in developing effective psychological interventions and media literacy programs. However, the artificial nature of experimental environments can limit the generalizability of the findings to real-world settings, where the interplay among myriad factors is more complex and less controllable [202].

Collectively, these quantitative approaches offer a comprehensive toolkit for researchers, combining the broad reach of surveys with the depth of experimental methodologies to explore the significant effects of body image on psychological well-being. This dual approach not only advances our understanding of body image issues but also supports the development of targeted strategies to address them effectively.

### 8.2. Qualitative Research Methods

In-depth interviews and focus groups serve as critical qualitative methods in body image research, each offering unique insights into the subjective experiences of individuals and groups, respectively. In-depth interviews provide a detailed exploration of individual perceptions of body image, delving into the emotional and psychological impacts of these perceptions and how they are influenced by societal and cultural norms. This method excels in uncovering the layers of personal meaning, belief systems, and internalized standards that drive body image satisfaction or dissatisfaction [203]. Interviews can reveal intricate details about how body image intersects with identity, self-esteem, and mental health, offering a rich understanding of individual experiences. However, the qualitative nature of interview data poses challenges in generalization, as findings are deeply tied to the specific participants involved. Furthermore, the sensitivity of body image topics requires skilled interviewing to ensure a comfortable and empathetic environment, which can influence the consistency and reliability of the data collected [204]. Focus groups, on the other hand, provide a dynamic environment where the social dimensions of body image can be explored. Through group interactions, this method allows for the examination of collective attitudes, shared experiences, and common pressures surrounding body image. Focus groups are particularly effective in illuminating how societal expectations and peer influences shape body image perceptions, offering insights into the communal aspects of these issues [205]. However, the group setting can also introduce variables that affect the data, such as the influence of dominant voices or participants’ reluctance to share personal views in a public forum. These dynamics necessitate careful moderation to capture a broad range of experiences and perspectives accurately [206].

Together, both approaches enrich our understanding of the complex ways in which individuals and groups navigate body image issues. By integrating these qualitative methods with quantitative approaches, researchers can achieve a more holistic view of body image influences, supporting the development of targeted interventions and promoting a deeper understanding of how cultural and social contexts shape body image perceptions and psychological well-being.

### 8.3. Mixed Methods Approaches

The mixed methods approach in body image research offers a robust framework that combines the comprehensive scope of quantitative methods with the detailed insight of qualitative techniques, effectively addressing the multifaceted nature of body image issues. This integrative methodology not only broadens the understanding of the prevalence and patterns of body dissatisfaction but also deepens the exploration of the personal experiences that underlie these patterns [155].

Quantitative tools such as surveys and questionnaires serve to map out the landscape of body image satisfaction or dissatisfaction across varied demographics, pinpointing trends and associations with psychological outcomes like depression or self-esteem levels. These data provide a critical foundation for identifying specific groups or contexts where body image concerns are most acute, guiding the direction of further qualitative inquiry. Subsequent qualitative methods, such as in-depth interviews or focus groups, build on this quantitative base by delving into the personal narratives that statistical data alone cannot capture. These discussions can uncover the rich emotional, cultural, and social nuances influencing individuals’ perceptions of their bodies [168]. They explore the impacts of media influence, societal pressures, and personal health factors on body image, offering a window into the lived experiences of individuals affected by body dissatisfaction. By employing a mixed methods approach, researchers can capture a comprehensive understanding of body image issues. This methodology not only identifies and quantifies the extent of body image concerns but also contextualizes these findings within the real-world experiences of individuals, providing a layered understanding that is vital for developing effective interventions and support mechanisms. This dual approach ensures that interventions are not only statistically validated but are also grounded in the actual needs and circumstances of those they aim to help, facilitating more tailored and impactful health promotion efforts [207].

### 8.4. Longitudinal Studies

Longitudinal studies are invaluable in the realm of body image research, providing unique insights into how perceptions of body image evolve over time and across different life stages. By tracking the same individuals or cohorts over extended periods, these studies allow researchers to observe the natural progression of body image satisfaction or dissatisfaction and to identify critical factors that influence changes in these perceptions [207]. For instance, longitudinal research can examine the effects of pivotal life events, such as puberty, pregnancy, or menopause, on body image. It can also assess how cultural shifts and evolving societal standards impact individuals’ views of beauty and self-worth over many years. The strength of longitudinal studies lies in their ability to establish temporal sequences and causal relationships, distinguishing transient body image concerns from those that are more enduring. This long-term perspective is crucial for understanding the persistent or changing nature of body image issues and their prolonged effects on mental health [128].

Despite their extensive benefits, longitudinal studies require considerable resources, including a sustained commitment to participant follow-up, meticulous data collection, and rigorous analysis over potentially decades. The logistical and financial demands of these studies are significant, but the depth and richness of the data they provide make them an essential component of body image research. The findings from longitudinal studies not only enhance our understanding of the development and trajectory of body image concerns but also inform the creation of targeted interventions designed to promote body positivity and psychological resilience throughout various phases of life.

### 8.5. Strengths and Limitations

The diversity of methodological approaches in body image research underscores the complexity of the subject and the varied dimensions it encompasses. Each methodology offers unique advantages and faces distinct challenges in capturing the nuances of body image and its impacts on psychological well-being.

Quantitative methods, such as surveys and experiments, provide broad, generalizable data that can reveal patterns and correlations across large populations. They are instrumental in quantifying the extent of body image issues and their association with psychological outcomes like anxiety or depression. However, these methods may fall short in capturing the depth and subjective experiences of individuals, as they often rely on standardized questions that might not reflect personal nuances. Moreover, qualitative methods, including in-depth interviews and focus groups, offer detailed insights into the personal and emotional aspects of body image. They allow researchers to explore the rich, contextual narratives that define individual experiences, revealing how cultural, social, and personal factors intertwine to influence body perception. While immensely valuable, these approaches typically involve smaller sample sizes, which may limit their generalizability across broader populations. In addition, mixed methods research seeks to integrate the strengths of both quantitative and qualitative approaches, providing a more comprehensive understanding of body image issues. By combining numerical data with personal narratives, mixed methods studies can contextualize the statistical trends within the lived experiences of individuals, offering a balanced view that enhances both the depth and breadth of the findings. Furthermore, longitudinal studies, with their extended follow-up periods, are crucial for tracing the evolution of body image perceptions over time. They are uniquely positioned to document how individual and societal changes impact body image, providing insights into causality and long-term trends. However, these studies require significant time and resource commitments, which can be a major limitation.

In conclusion, the variety of methodological approaches in body image research reflects the need for a multifaceted analysis to grasp fully how these perceptions impact psychological health. Each method has its strengths and limitations, but together, they form a robust toolkit that can address the intricate dynamics of body image across different contexts and timeframes. This methodological diversity is crucial for developing targeted, effective interventions and policies that address the wide spectrum of body image issues faced by individuals worldwide.

## 9. Interventions and Strategies for Improving Body Image

### 9.1. Interventions and Strategies 

The imperative to enhance body image satisfaction intersects crucially with public health and psychological well-being. Given the complex influences of cultural, social, and individual factors on body image, there exists a pressing need to develop and implement robust interventions [166]. Such endeavors require the concerted efforts of healthcare professionals, educators, policymakers, and the broader community to forge a healthier societal relationship with body image. This section details a variety of effective interventions, underscoring the vital roles various stakeholders play in promoting a positive and inclusive view of body image across diverse societal segments [168].

### 9.2. Prevention and Intervention Strategies

Enhancing body image satisfaction is essential for public health and psychological well-being, necessitating collaborative interventions from various sectors. These strategies can be grouped into prevention and intervention categories, and further classified into universal, targeted, and indicated interventions.

#### 9.2.1. Universal Interventions

Media Literacy Programs:

Media literacy programs educate especially the youth about the realities behind media portrayals, fostering a critical understanding of beauty standards and reducing the impact of negative media influences.

Educational System Programs:

Implementing body positivity and self-esteem enhancement programs within educational systems helps nurture healthy body image from a young age. These programs discuss the impacts of media, the diversity of body types, and the importance of appreciating body functionality over appearance, thus playing a pivotal role in challenging societal beauty norms and promoting inclusivity [208].

Public Health Campaigns:

Legislators and policymakers play a critical role in promoting a healthier body image across society by enacting policies that regulate the portrayal of body standards in media and supporting public health campaigns that celebrate body diversity. These efforts contribute to a societal framework that supports positive body image and psychological well-being for all individuals.

#### 9.2.2. Targeted Interventions

Cognitive Behavioral Therapy (CBT):

Cognitive behavioral therapy is vital in this effort, as it helps individuals unravel negative perceptions and adopt healthier attitudes toward their bodies [206].

Mindfulness and Self-Compassion Practices:

Mindfulness and self-compassion practices also significantly enhance body satisfaction by fostering kindness and understanding toward oneself, helping individuals navigate body image concerns with less self-judgment.

Healthcare Professional Involvement:

Healthcare professionals are crucial in detecting and managing body image issues, advocating for a shift in healthcare focus from weight to overall health and well-being. This approach promotes a more compassionate healthcare environment that emphasizes health outcomes over numerical weight goals [209].

#### 9.2.3. Indicated Interventions

Clinical Interventions for Severe Cases:

For those battling significant body image dissatisfaction, mental health interventions like CBT, acceptance and commitment therapy, and dialectical behavior therapy can be effective. These therapies help individuals manage distressing thoughts and emotions related to body image, enhancing psychological resilience [45].

Support Groups:

Establishing support groups provides a supportive community environment where individuals can share experiences and strategies for coping with body image issues. This collective support is vital for fostering a sense of belonging and mutual encouragement.

#### 9.2.4. Multisectoral Collaboration

Addressing the multifaceted challenge of promoting positive body image requires a collaborative effort that spans multiple sectors, including healthcare, education, policymaking, media, and the community. This endeavor involves dismantling entrenched societal norms, building resilience against pervasive media influences [210], and nurturing environments that celebrate diversity and individuality. Implementing evidence-based interventions and adopting a collaborative approach enhances the potential for meaningful change, fostering a future where body image satisfaction is accessible to everyone [211].

Healthcare professionals, educators, policymakers, media creators, and community leaders each play a pivotal role in this process. By adopting a holistic perspective that recognizes the interplay among cultural, social, media, and individual factors, stakeholders can develop effective strategies to address core body image issues [212]. This includes advocating for policies that ensure diverse and realistic media representations, creating educational curricula that empower young people to navigate body image challenges confidently, and providing accessible mental health services to address body dissatisfaction and its psychological impacts. Moreover, fostering community-wide dialogues about body image, challenging stigmatizing behaviors, and promoting acceptance can contribute to a cultural shift toward greater body positivity [213,214,215].

Promoting positive body image is a continuous process that demands resilience, innovation, and collaboration. By leveraging the strengths of various sectors and embracing a comprehensive approach, we can make significant strides toward improving body image satisfaction. These efforts enhance not only the psychological well-being of individuals but also contribute to the health and vitality of the broader community. As we persist in advocating for change, the vision of a society where every individual can experience a positive and affirming relationship with their body becomes increasingly attainable, marking a significant step toward holistic health and well-being.

## 10. Policy Implications and Future Directions

These findings highlight the complexity of body image issues and how various factors intertwine to influence individuals’ perceptions and mental health. This exploration provides valuable insights for health policy and practice, suggesting that interventions and public health strategies need to be nuanced and multifaceted to address the spectrum of body image concerns effectively. Furthermore, this review identifies key areas ripe for future research, pointing out gaps in our current understanding and emphasizing the dynamic nature of body image issues amid societal and technological changes. The implications of these findings are substantial, affecting healthcare providers, educators, policymakers, and individuals—all integral to creating environments that promote positive body image and psychological well-being. As we address the complexities identified in this review, it is evident that a collaborative, interdisciplinary approach is required, one that combines insights from psychology, sociology, public health, and other fields. By building on this foundation and exploring the identified areas for further research, we can develop and refine intervention strategies that are both effective and empathetic. Ultimately, our goal is to transform body image satisfaction from an aspiration to a reality for individuals across diverse populations, thereby enhancing the overall psychological well-being of our communities.

### 10.1. Implications for Health Policy and Practice

The interplay between body image and mental health is a critical area for policy intervention and practical application in healthcare and education. Given the pervasive influence of media on body image, policymakers are urged to enforce regulations that require advertisers and media outlets to disclose when images have been digitally altered. This transparency is key in helping individuals recognize the discrepancy between real human bodies and the often unrealistic standards portrayed in the media, which can significantly mitigate body dissatisfaction and its associated mental health repercussions, such as depression and anxiety.

In the educational sector, integrating body image and media literacy into school curricula is imperative. Teaching students to critically evaluate the media’s portrayal of beauty and body standards empowers them to embrace body positivity, enhancing their self-esteem and fostering a more inclusive school environment. Supporting this educational shift through health policies can have a lasting impact on youth mental health and well-being. Healthcare professionals also play a vital role in addressing body image issues. Policies should support specialized training that emphasizes a holistic approach to health, prioritizing functionality and health over appearance. This training is crucial for dismantling weight-related stigmas and enabling healthcare providers to deliver care that is compassionate and supportive of both mental and physical health. Lastly, supporting research into body image issues is essential for developing effective interventions. Policymakers should allocate funds to study the psychological impact of media and new technologies like social media on body image. Such research can inform targeted interventions that promote healthy body image perceptions and overall mental wellness across diverse communities. By grounding these policies in robust research, we ensure that interventions are evidence-based and tailored to address the nuanced challenges of body image dissatisfaction effectively.

### 10.2. Suggestions for Future Research

The necessity for sophisticated research methodologies to delve deeper into the complexities of body image and its psychological implications is highlighted by ongoing societal and technological changes. Longitudinal studies are essential as they provide insights into the long-term effects of social media on body image and psychological well-being, helping to discern causal relationships and factors that may be modified over time. This is crucial for developing effective preventive measures and informed policy decisions in mental health care. Additionally, future research must prioritize inclusivity by incorporating a wide range of demographics such as different ages, genders, cultural backgrounds, and socioeconomic statuses. Ensuring diversity in research participants guarantees that the findings are comprehensive and applicable across various societal segments, which is vital for crafting equitable and effective interventions and policies.

There is also a significant opportunity to evaluate the efficacy of interventions aimed at improving body image satisfaction. Studies should focus on assessing the impact of educational programs, digital tools, and community-based initiatives to determine which are most effective across different settings. This will allow stakeholders to direct resources toward the most impactful and cost-efficient strategies. Moreover, with the rapid advancement of technologies like virtual reality, augmented reality, and AI-driven algorithms, it is imperative to investigate how these innovations influence body image and overall psychological health. Research in this area will provide essential guidance for technology developers and policymakers to mitigate potential negative effects. Furthermore, exploring how gender and cultural factors influence body image and psychological health is critical for developing targeted interventions. Different cultural and gender groups experience and internalize body image issues in unique ways. Research that addresses these nuances can lead to more personalized and culturally sensitive approaches, ensuring that interventions are respectful and relevant to the specific needs of diverse populations. By tackling these areas, researchers and practitioners can enhance their understanding of body image issues and develop more effective interventions, paving the way for improved body image satisfaction and psychological well-being across the broader community.

## 11. Conclusions

This review delves into the complex interplay among body perceptions, social media, physical metrics, and their combined effects on psychological well-being, focusing particularly on body image satisfaction and its links to cultural and gender dynamics. It synthesizes findings from a range of studies to shed light on how these factors influence self-esteem, mental health, and overall life satisfaction. The analysis underscores the profound influence of societal standards and media portrayals on individual psychological states. By unpacking the nuanced ways in which these elements interact, this review enhances our understanding of the far-reaching impact of external influences on personal health. It highlights the need for targeted interventions and thoughtful policies that are informed by a deep comprehension of these complex relationships. Moving forward, it is critical to continue this exploration to ensure that future strategies and research are rooted in a well-rounded perspective, aiming to bolster psychological well-being across diverse groups.

## Figures and Tables

**Table 1:** Body perception psychological outcomes.

Positive Psychological Outcomes	Negative Psychological Outcomes
Lower rates of depression and anxiety Enhanced self-esteem Improved quality of life Healthier sexual functioning	Eating disorders Depression and low self-esteem Unhealthy behaviors Social avoidance

## Data Availability

Not applicable.
